# Renin–angiotensin system activation and oxidative stress in hospitalized COVID-19 patients: a single-centre prospective observational study

**DOI:** 10.1186/s40635-026-00857-w

**Published:** 2026-02-10

**Authors:** Davide Eleuteri, Filippo Del Tedesco, Federico Silvia, Caterina Tucciariello, Ersilia Ruggiero, Jacopo Gervasoni, Lavinia Santucci, Aniello Primiano, Martina Petrucci, Ernico Torelli, Rossella Cianci, Diana Giannarelli, Salvatore Lucio Cutuli, Gennaro De Pascale, Giuseppe Bello, Calabrese Maria, Matteo Masullo, Andrea Urbani, Riccardo Di Santo, Massimo Antonelli, Luca Montini, the Gemelli-ICU Study Group, Alessandra Bisanti, Simone Carelli, Paolo De Santis, Valentina Di Gravio, Valentina Giammatteo, Domenico Luca Grieco, Antonio Gullì, Daniel Livanu, Rikardo Xhemalaj, Giovanna Mercurio, Gabriele Ciasca

**Affiliations:** 1 Department of Intensive Care and Anesthesiology, Fondazione Policlinico Universitario A. Gemelli IRCCS, Rome, Italy; 2 Catholic University of Sacred Heart, Rome, Italy; 3 Department of Basic Biotechnological Sciences, Intensivological and Perioperative of Basic Biotechnological Sciences, Intensivological and Perioperative Clinics, Fondazione Policlinico Universitario Agostino Gemelli IRCCS, Rome, Italy; 4 Dipartimento Di Emergenza-Urgenza, Fondazione Policlinico Universitario A. Gemelli IRCCS, Rome, Italy; 5 Departement of Traslational Medicine and Surgery, Fondazione Policlinico Universitario A.Gemelli, IRCSS, Rome, Italy; 6 Facility of Epidemiology and Biostatistics, Fondazione Policlinico Universitario Agostino Gemelli IRCCS, Rome, Italy; 7 Department of Life Science Health and Health Professions, Link Campus University Rome, Rome, Italy

**Keywords:** Renin–angiotensin system, Oxidative stress, Asymmetric dimethylarginine, COVID-19, Endothelial Activation and Stress Index, Worsening respiratory status, Personalized treatment strategies, Cardiovascular–kidney–metabolic syndrome, Angiotensin-converting enzyme 2, World Health Organization ordinal scale

## Abstract

**Background:**

The renin–angiotensin system (RAS) plays a critical role in vascular homeostasis and inflammation, and its dysregulation has been implicated in the pathophysiology of COVID-19. We investigated the dynamics of RAS peptides and markers of endothelial dysfunction in relation to respiratory disease progression in hospitalized COVID-19 patients.

**Methods:**

In this single-centre prospective observational study, 155 adult patients with confirmed SARS-CoV-2 infection were enrolled at hospital admission. Plasma levels of renin, angiotensin I (Ang I), angiotensin II (Ang II), angiotensin 1–7 (Ang 1–7), the Ang II/Ang I ratio (as a surrogate of ACE activity), and asymmetric dimethylarginine (ADMA)—a marker of endothelial dysfunction —were measured at baseline (D0) and day 3 (D3). Endothelial injury was further assessed using the Endothelial Activation and Stress Index (EASIX). Patients were stratified by respiratory trajectory using the WHO ordinal scale. Biomarker kinetics were analysed with baseline-adjusted regression models, and 28-day clinical status was evaluated using partial proportional-odds regression.

**Results:**

Of 155 patients, 89 (57%) experienced worsening respiratory status. These patients exhibited progressive RAS activation with higher renin and Ang I at D3 (*p* < 0.001), a decline in the Ang II/Ang I ratio (*p* < 0.001), and a rise in Ang 1–7 (*p* < 0.001). ADMA and EASIX levels increased in parallel, with significantly higher ADMA in worsening vs. non-worsening patients at D3 (0.72 [0.62–0.87] vs. 0.61 [0.50–0.70] µM/L; *p* < 0.001). Biomarker trajectories differed according to disease course, with significant interaction terms between baseline values and respiratory deterioration. At 28 days, outcomes were associated with renin, Ang I, Ang 1–7, and the Ang II/Ang I ratio, but not with Ang II. Elevated baseline ADMA also independently predicted worse prognosis.

**Conclusions:**

Worsening respiratory status in COVID-19 is associated with delayed activation of the RAS, a shift toward the alternative Ang 1–7 pathway, and parallel increases in endothelial dysfunction markers. These findings suggest that serial measurements of RAS peptides and ADMA may aid in identifying high-risk phenotypes and inform personalized therapeutic strategies in COVID-19.

**Supplementary Information:**

The online version contains supplementary material available at 10.1186/s40635-026-00857-w.

## Background

The renin–angiotensin system (RAS) constitutes a complex regulatory mechanism that plays a pivotal role in cardiovascular and fluid homeostasis, influencing numerous adaptive physiological processes. This system, composed of various peptides, enzymes, and receptors, enables finely tuned responses to physiological stress [1, 2]. Its principal effector, angiotensin II (Ang II), is produced through the cleavage of angiotensin I (Ang I) by endothelial angiotensin-converting enzyme (ACE), following the classical RAS pathway. Ang II not only modulates vascular tone and blood pressure, but is also involved in inflammatory response, cellular proliferation, and oxidative stress [3]. Recent research has highlighted the role of angiotensin-converting enzyme 2 (ACE2), which converts Ang II into the vasodilatory peptide angiotensin 1–7 (Ang 1–7), forming a counterregulatory axis within the RAS. Together with the Mas receptor, ACE2 and Ang 1–7 constitute the alternative arm of the system, characterized by anti-inflammatory and antioxidative properties [4, 5]. While an imbalance between ACE and ACE2 activity has been linked to a wide range of chronic cardiovascular diseases [6, 7], preclinical studies also suggest that RAS dysregulation may contribute to the pathophysiology of pulmonary inflammation [8–11]. The discovery of ACE2 as a functional receptor for SARS-CoV-2 has prompted numerous studies examining the quantitative dynamics of RAS peptides during COVID-19, though findings have been inconsistent [12–16]. Clinical and histological evidence indicate that SARS-CoV-2 infection directly impacts vascular endothelial cells, resulting in endothelitis and thrombotic events [17, 18]. From this perspective, vascular injury may be a key driver of COVID-19 outcomes and could help explain the increased morbidity and mortality observed in patients with pre-existing cardiovascular conditions [19, 20]. Not surprisingly, asymmetric dimethylarginine (ADMA) —a biomarker of vascular oxidative stress and a recognized indicator of cardiovascular risk—has been validated as a predictor of COVID-19 outcomes [21, 22]. Collectively, the available evidence supports a biologically plausible model, wherein endothelial injury, in conjunction with RAS dysregulation, may underlie the development of severe COVID-19 manifestations. To further explore this hypothesis, we analyzed plasma concentrations of RAS components and ADMA in patients with COVID-19 across a spectrum of disease severity. Measurements were conducted throughout hospitalization, with the objective of identifying potential biomarkers to aid in patient stratification and guide targeted therapeutic interventions.

## Methods

### Study design and clinical setting

This was a prospective observational study involving patients admitted to the Emergency Department at Fondazione Policlinico Universitario A. Gemelli IRCCS (Rome, Italy) between April and September 2021. All consecutive adult patients (≥ 18 years) presenting with epidemiological risk and clinical features consistent with SARS-CoV-2 infection were screened. Specifically, all screened patients had a positive SARS-CoV-2 antigenic test and at least one of the following: fever, respiratory symptoms, or radiological evidence of pneumonia/viral pneumonitis. Inclusion criteria were (a) provision of informed consent; (b) confirmed SARS-CoV-2 infection as determined by real-time reverse transcriptase-polymerase (RT-PCR); (c) an expected hospital stay of more than 48 h; and (d) feasibility of sampling within the first 24 h after hospital admission. Exclusion criteria included pregnancy and moribund state. The study protocol was approved by the local Ethics Committee. All patients, or their legal representatives where applicable, provided written informed consent prior to inclusion in the study, in accordance with national law and the Declaration of Helsinki. The study design and manuscript preparation adhered to the Strengthening the Reporting of Observational Studies in Epidemiology (STROBE) guidelines (Supplementary Methods) [23].

### Timeline and measurements

Blood samples were collected from each participant at two timepoints at enrollment (D0)—within 24 h of hospital admission—and on day 3 (D3). Sample collection was coordinated with clinically indicated blood draws. Analytical techniques for quantifying plasma levels of RAS peptides and ADMA are described in detail in the Supplementary Methods. Briefly, serum renin was measured using a chemiluminescent immunoassay, whereas angiotensin peptides (Ang I, Ang II, Ang 1–7) were quantified using a steady-state ultraperformance liquid chromatography–tandem mass spectrometry (UPLC–MS/MS) method. ADMA was measured using UPLC–MS/MS following standard deproteinization procedures. The ratio of angiotensin II to angiotensin I concentration (Ang II/I) was calculated as a surrogate marker of ACE activity. The clinical course was monitored for 28 day post-enrollment or until discharge, whichever occurred later. Additional methodological details, including demographic and laboratory parameters, criteria for organ dysfunction, and definitions of comorbidities, such as cardiovascular–kidney–metabolic (CKM) syndrome, are also provided in the Supplementary Methods. RAS medication status was defined as treatment with a pharmacologic RAS inhibitor at the time of hospitalization. Markers of endothelial dysfunction comprised ADMA and the Endothelial Activation and Stress Index (EASIX), which was calculated using the following formula: EASIX = LDH (U/L) × creatinine (mg/dL) ÷ platelet count (10⁹/L) [24]. Respiratory status was graded using a modified WHO ordinal scale [25], in which higher values indicate lower levels of respiratory support (better clinical status) and lower values indicate higher levels of support (worse clinical status). Respiratory trajectories were defined by comparing the scale value at day 3 (D3) with that at baseline (D0). Patients were classified into two groups: (i) worsening, if the D3 score was lower than the D0 score (D3 < D0), and (ii) non-worsening, if the D3 score was equal to or higher than the D0 score (D3 ≥ D0).

### Main objective

The main objective of this study was to describe the relationship between RAS peptide concentrations and markers of endothelial damage with the trajectory of respiratory support level in hospitalized patients with COVID-19.

### Endpoints

The primary endpoint was to assess whether changes in WHO ordinal scale levels were associated with variations in RAS peptide and ADMA plasma concentrations during the study period. The secondary exploratory endpoints were: (1) to evaluate the relationship between RAS peptide concentrations and markers of endothelial injury; and (2) to evaluate the association of RAS peptides and ADMA at both timepoints with the 28-day clinical outcome.

### Statistical methods and power analysis

Descriptive statistics were used to summarize the data: categorical variables are presented as absolute counts and percentages, and continuous variables as median with interquartile range. Analyses were performed using Stata, version 18 (StataCorp LLC, College Station, TX); tables and figures were generated in R, version 3.4.4 (https://www.r-project.org). Sample size calculations were based on a multiple linear regression model testing the joint effect of two predictors (partial $F$ test, numerator df = 2) while adjusting for six covariates (total predictors = 8). Assuming a medium effect size (f^2^ = 0.10), a Bonferroni-corrected α = 0.008 (for six planned models), and 80% power, the required sample size was approximately *N* = 150 (denominator df ≈*N* − 9). Missing data were addressed using the multiple imputation by chained equations (MICE) algorithm, with predictive mean matching (PMM) technique to account for non-normally distributed continuous variables [26]. The primary objective was assessed using baseline-adjusted linear regression models. For each of the six biomarkers, a separate model was constructed with the D3 plasma biomarker level as the dependent variable. Covariates included the corresponding D0 biomarker value, age, and the Sequential Organ Failure Assessment (SOFA) score at inclusion. Patient group (worsening vs. non-worsening), sex, chronic use of RAS inhibitors, and history of CKM syndrome were included as categorical variables. Each model also included an interaction term between the D0 biomarker value and patient group. Because residual diagnostics suggested heteroskedasticity, inference was based on nonparametric bootstrap (2000 replications) and confirmed using heteroskedasticity-robust (HC3) standard errors [27]. Variable selection was guided by both statistically significant findings in univariate analyses (*p* < 0.20) and clinical relevance supported by the literature [28]. For each model, Wald test $p$ values were reported for the patient group and the interaction term. The discriminative performance of RAS peptides and ADMA for predicting invasive ventilation or death at D3 was assessed using receiver operating characteristic (ROC) analysis. In addition, generalized ordered logit (gologit) models were applied to evaluate the association of each biomarker, at both timepoints, with 28-day clinical status. Clinical status was categorized into three levels: 1 = deceased or receiving invasive mechanical ventilation (IMV); 2 = hospitalized but not requiring IMV; 3 = alive and discharged from the hospital. Biomarkers were standardized to facilitate interpretation; thus, odds ratios reflect the effect of a one–standard deviation (SD) increase in biomarker level on the odds of worse 28-day clinical status. Models were adjusted for age, sex, history of CKM syndrome, and SOFA score at inclusion. RAS inhibitor use was excluded from the primary models owing to the limited number of patients in some outcome categories and its weaker association with 28-day clinical status in the initial univariate screen. However, to further examine potential confounding, we repeated analyses, including RAS inhibitor use. Multiplicity across gologit models for the six biomarkers was addressed with the Benjamini–Hochberg (BH) procedure, controlling the false discovery rate (FDR) at 5% (Supplementary Methods).

## Results

### Clinical characteristics of study population

**Fig. 1:**
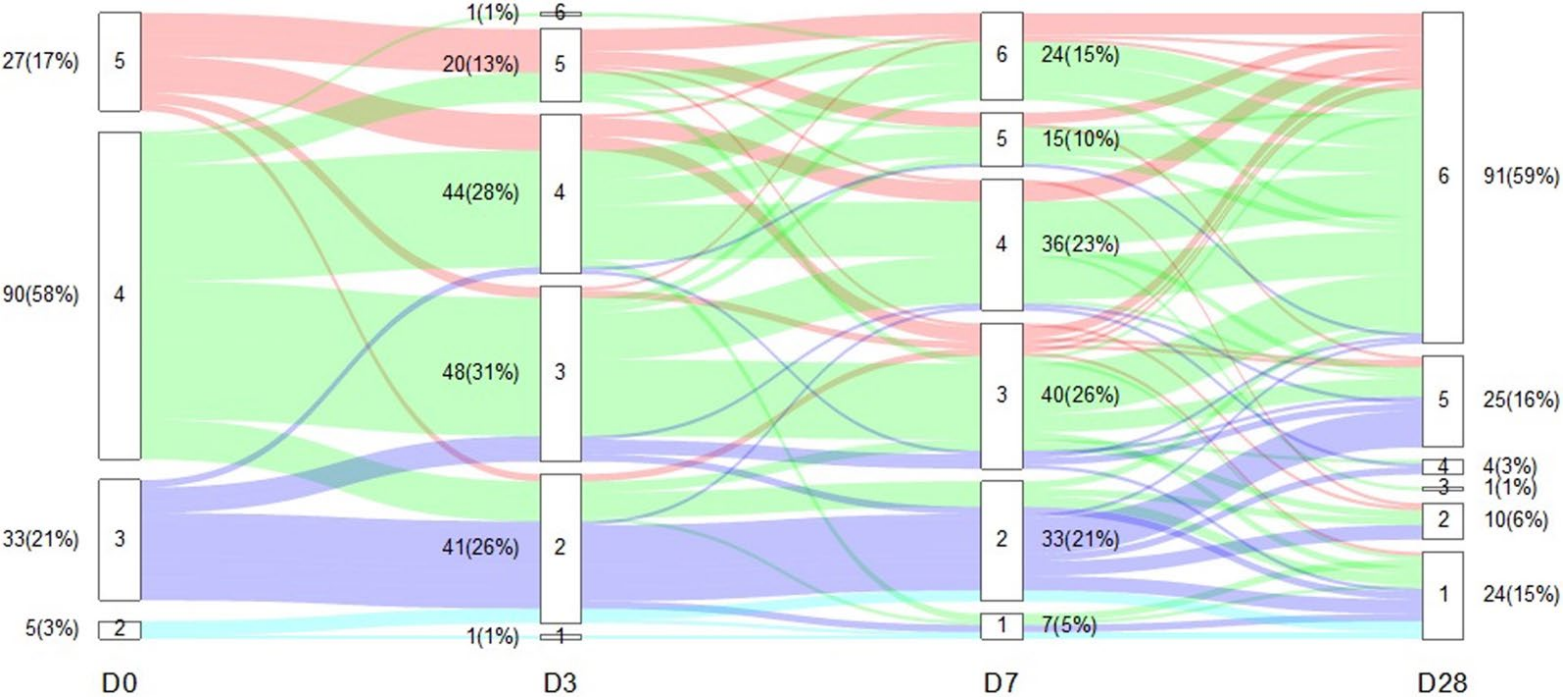
Longitudinal distribution of patients according to the World Health Organization (WHO) ordinal scale. The lines represent the redistribution or stability of patients over time across the levels of the World Health Organization (WHO) clinical progression scale. D0 = day 0; D3 = day 3; D7 = day 7; and D28 = day 28, relative to study inclusion. Levels of respiratory support according to the modified WHO scale: [1]: deceased; [2]: invasive mechanical ventilation (IMV); [3]: non-invasive mechanical ventilation (NIMV) or high-flow nasal cannula (HFNC); [4]: conventional oxygen therapy (COT); [5]: hospitalized without oxygen supplementation; [6]: alive and discharged from the hospital

A total of 155 patients were included, of whom 66 (43%) were in the non-worsening and 89 (57%) in the worsening group (Fig. 1 and e-Fig. 1; Table 1 and e-Table 1). The median age was 61 years, and one-third were female; baseline demographics were similar between groups. Compared with the non-worsening group, patients in the worsening group had a lower PaO₂/FiO₂ ratio and higher SOFA score at admission (*p* < 0.001 for both). CKM syndrome and prior use of RAS inhibitors were more frequent in the worsening group (*p* = 0.005 and *p* = 0.004, respectively). In-hospital outcomes differed: worsening patients more often required intensive care unit (ICU) admission and invasive ventilation and had longer hospital stays (all *p* < 0.001).

**Table 1:** Clinical characteristics of the study cohort

Variable	All patients (*n* = 155)	Non-wors (*n* = 66)	Wors (*n* = 89)	*p* value
Age (years)	61 (52–74)	62 (51–75)	60 (52–72)	0.49(†)
Female, n (%)	50 (32.3)	22 (33.3)	28 (31.5)	0.80(††)
Man, n (%)	105 (67.7)	44 (66.7)	61 (68.5)	
BMI Kg/m^2^ (IQR)	26.2 (24.7–29.4)	26.1 (24.5–28.2)	27.7 (24.7–29.4)	0.16(†)
CKM syndrome, n (%)	88 (56.8)	29 (43.9)	59 (66.3)	0.005(††)
RAS-inhibitor use, n (%)	42 (27)	10 (15)	32 (36)	0.004(††)
Characteristics at enrolment				
Symptoms onset to inclusion, days (IQR)	7 (5–10)	7 (5–10)	7 (4–9)	0.21(†)
PaO₂/FiO₂ mmHg (IQR)	238 (147–310)	310 (238–361)	209 (108–281)	< 0.001(†)
Without oxygen supplementation, n (%)	27 (17.4)	13 (19.7)	14 (15.7)	0.52(††)
SOFA score	2 (2–3)	2 (1–3)	2 (2–5)	< 0.001(†)
Creatinine (mg/dl)	0.9 (0.7–1.1)	0.84 (0.67–1.05)	0.92 (0.74–1.20)	0.11(†)
C-reactive protein (mg/dl)	68 (32–119)	59.9 (28.5–103.2)	72.5 (39.4–137.5)	0.02(†)
Absolute Lymphocyte count (10⁹/l)	0.7 (0.5–1)	0.73 (0.51–1.14)	0.69 (0.49–0.93)	0.37(†)
Absolute White Cell count (10⁹/l)	8.4 (6–10)	7.42 (5.42–9.24)	8.77 (6.30–10.26)	0.07(†)
Platelet count (10⁹/l)	220 (165–291)	217 (174–295)	226 (157–289)	0.73(†)
D-dimer (ng/ml)	900 (500–1625)	798 (729–871)	969 (566–1275)	0.022(†)
Outcome				
IMV during hospitalization, n (%)	42 (51.8)	6 (9.1)	36 (40.4)	< 0.001(††)
ICU admission, n (%)	81 (52.2)	15 (22.7)	66 (74.1)	< 0.001(††)
AKI during hospitalization, n (%)	25 (16.1)	6 (9.1)	19 (21.3)	0.04(††)
Hospital stays (days)	14 (8–26)	9 (6–14)	20 (11–32)	< 0.001(†)
Hospital mortality, n (%)	34 (21.9)	11 (16.7)	23 (25.8)	0.17(††)
28-day clinical status, n (%)				
Dead or on IMV	34 (21.9)	12 (35.3)	22 (64.7)	0.04(††)
Hospitalized not requiring IMV	30 (19.4)	8 (26.7)	22 (73.3)	
Alive and discharge from the hospital	91 (58.7)	46 (50.5)	45 (49.5)	

*n*  number of patients, *Non-wors*  non-worsening group; *Wors*  worsening group, *BMI*  body-mass index, *CKM*  cardiovascular–kidney––metabolic, *RAS*  Renin–angiotensin system, RAS medication status was defined as treatment with a pharmacologic RAS inhibitor at the time of hospitalization. *PaO₂*  partial pressure of arterial oxygen; *FiO₂*  fraction of inspired oxygen, *SOFA*  Sequential Organ Failure Assessment score, *IMV*  invasive mechanical ventilation, *AKI*  Acute Kidney Injury. Variables were presented as median with interquartile range unless otherwise indicated. (†) = Mann–Whitney test; (††) = Chi-Square

### Worsening patients show RAS activation with a shift toward the alternative pathway

Overall, we analyzed 310 samples (e-Table 2). Table 2 reports the median systemic levels of the biomarkers studied, stratified by group. Worsening patients exhibited progressive RAS activation, with D3 levels of renin and Ang I significantly higher compared to non-worsening patients (64.4 [IQR 20.4–203.6] vs. 9.2 [IQR 4.1–20.7] and 1211 [IQR 630.7–3465] vs. 191 [IQR 102.5–351] pg/mL; *p* < 0.001, respectively). While Ang II levels decreased at D3 in both groups, Ang II/Ang I, an estimation of ACE activity, displayed a significant decrease from D0 to D3 only in worsening group (*p* < 0.001). Unlike the other RAS peptides, Ang 1–7 concentrations at D0 were significantly lower in worsening than non-worsening group (18.8 [IQR 10–43] vs. 35 [IQR 21–59.5] pg/ml with *p* = 0.02, respectively), with this relationship reversed at D3 (69.5 [IQR 32–114] vs. 16 [IQR 10–33.5] pg/ml with *p* < 0.001, respectively). Consistently, renin and the Ang II/Ang I demonstrated a strong negative correlation at both D0 and D3 (*ρ* = − 0.65 and *ρ* = − 0.81; *p* < 0.001, respectively), while renin and Ang 1–7 were positively correlated at both timepoints (*ρ* = 0.37 and *ρ* = 0.74; *p* < 0.001, respectively) (e-Fig. 3). To exclude potential interference from RAS inhibitors therapy, we conducted a prespecified subgroup analysis restricted to patients not receiving RAS inhibitors at admission (*n* = 113). Biomarker trajectories in this subgroup (Table E9) reproduced the same patterns observed in the overall cohort, with worsening patients demonstrating progressive increases in renin and Ang I, a decline in the Ang II/Ang I ratio, and rising Ang 1–7 and ADMA levels. Notably, Ang II levels remained relatively stable and did not increase proportionally to upstream RAS activation, reproducing the blunted Ang II response seen in the full population. For all RAS peptides, the ability to discriminate between patients who required intubation or died by D3 was initially poor but improved over time. While the area under the ROC curve (AUC) at D0 exceeded 0.7 only for renin, the AUC at D3 was above 0.8 for renin, Ang I, Ang II/Ang I, and Ang 1–7 (Table 3).

**Table 2:** Marker systemic levels stratified by group at day 0 and day 3

		Day 0			Day 3	
Marker	Non-worsening	Worsening	p value^†^	Non-worsening	Worsening	p value^†^
Renin	14.9 (5.1–28.8)	18.9 (6.9–43.8)	0.13	9.2 (4.1–20.7)	64.4 (20.4–203.6)	< 0.001
Ang I	203 (100–365)	300.6 (113–766)	0.05	191 (102.5–351)	1211 (630.7–3465)	< 0.001
Ang II	19 (15–34)	24 (15.5–34.5)	0.02	16 (15–22.2)	20 (15–34.5)	0.10
Ang II/I	0.10 (0.04–0.15)	0.08 (0.04–0.16)	0.48	0.10 (0.05–0.15)	0.02 (0.01–0.03)	< 0.001
Ang1–7	35 (21–59)	18.8 (10–43)	0.02	17 (10–33)	69 (30.5–109.7)	< 0.001
ADMA	0.56 (0.45–0.63)	0.58 (0.50–0.76)	0.06	0.61 (0.49–0.70)	0.72 (0.62–0.87)	< 0.001
EASIX	1.31 (0.92–2.21)	1.63 (1.06–2.79)	0.22	0.90 (0.68–1.21)	1.06 (0.76–2.08)	0.01

Renin reported in pg/ml; *Ang*  angiotensin (pg/ml); *ADMA* = asymmetric dimethylarginine (µM/L); *EASIX* = endothelial activation and stress index. Values are reported as medians with interquartile ranges. † Mann–Whitney test (significance level < 0.05). *Ang II/I* = calculated as the ratio of angiotensin II to angiotensin I concentration

**Table 3:** RAS markers and ADMA discrimination for respiratory support level at day 3

		Day 0			Day 3	
Marker	AUC ± SE		95% CI	AUC ± SE		95% CI
Renin, pg/ml	0.70 ± 0.05		0.62–0.71	0.84 ± 0.03		0.77–0.89
Ang I, pg/ml	0.63 ± 0.05		0.55–0.71	0.84 ± 0.04		0.77–0.90
Ang II, pg/ml	0.56 ± 0.05		0.49–0.66	0.65 ± 0.06		0.57–0.73
Ang II/Ang I	0.59 ± 0.05		0.48–0.69	0.83 ± 0.04		0.76–0.89
Ang 1–7, pg/ml	0.53 ± 0.06		0.41–0.63	0.85 ± 0.03		0.77–0.90
ADMA, µM/L	0.66 ± 0.05		0.57–0.76	0.77 ± 0.04		0.69–0.84

Receiver operating characteristic (ROC) analysis evaluating the discriminative performance of RAS biomarkers and ADMA measured on day 0 and day 3 for identifying patients who, by day 3, either died or required invasive mechanical ventilation versus those who remained alive without invasive respiratory support. Area under the curve (AUC) values are reported with standard error (SE) and 95% confidence intervals (CI). *RAS* = Renin–angiotensin system. ADMA = Asymmetric dimethylarginine. *Ang* = angiotensin. *Ang II/I* = calculated as the ratio of angiotensin II to angiotensin I concentration

**Fig. 2:**
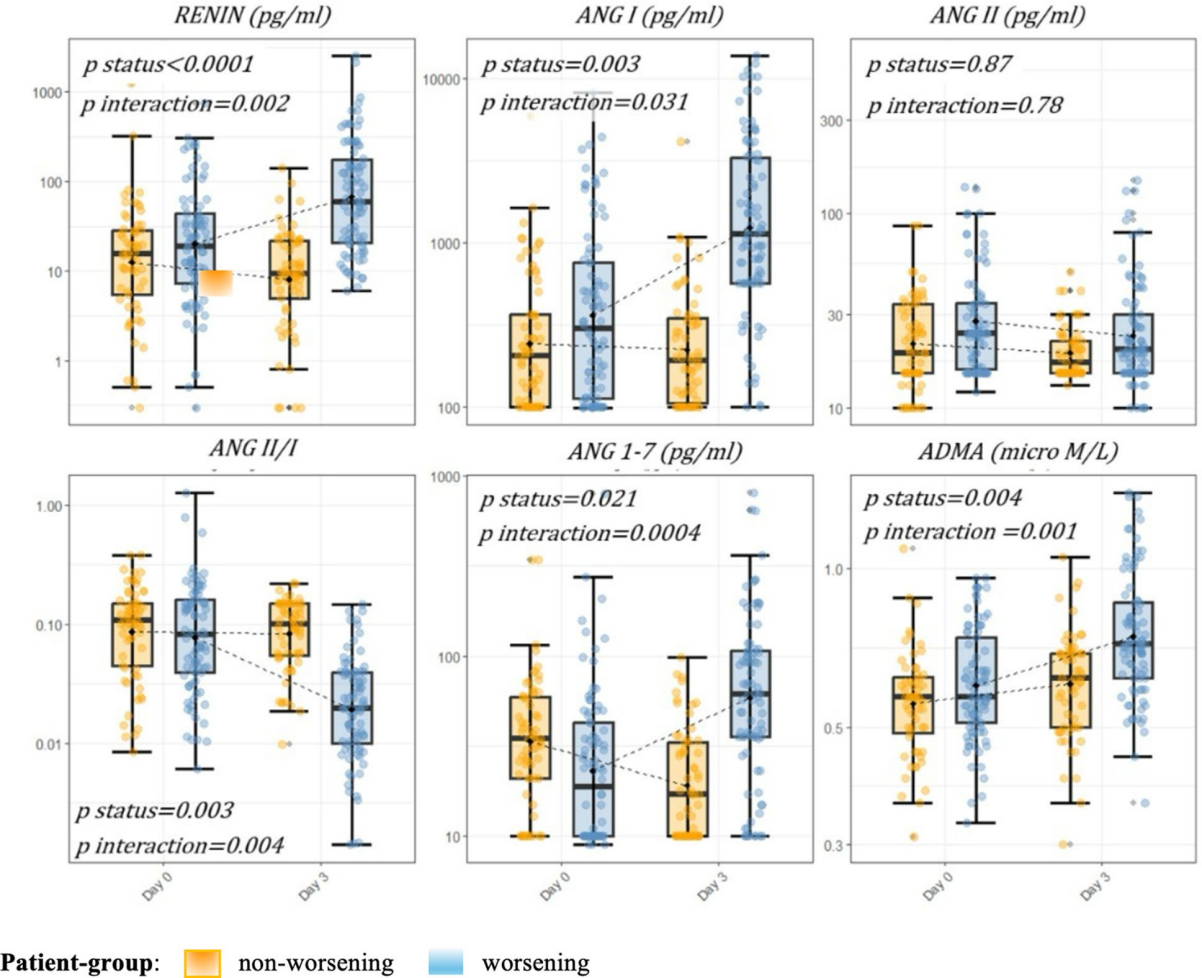
RAS markers and ADMA over time by patient group (worsening vs. non-worsening). RAS markers and ADMA over time by patient group (worsening vs. non-worsening). The $p$ values indicate the results of the statistical hypothesis tests from the multivariable linear regression models adjusted for day-0 biomarker, age, sex, sofa score at inclusion, cardiovascular–kidney–metabolic syndrome and use of RAS inhibitors at the time of hospitalization. These models evaluated differences in day 3 biomarker levels, accounting for patient group (*p* status) and the interaction between corresponding baseline marker levels of and patient group (*p* interaction). Each dot represents an individual patient. Box, 25th to 75th percentiles; whiskers, 5th to 95th percentiles. Dotted lines connect the means at each timepoint. *RAS*  Renin–angiotensin system, *Ang* = angiotensin, *ADMA*  asymmetric dimethylarginine

In statistical models, increases in plasma concentrations of renin, Ang I, and Ang 1–7 over the study period—but not Ang II—were associated with respiratory decline, as indicated by significant interaction terms between patient group and baseline biomarker levels (*p* ≤ 0.001 for each marker). In contrast, the Ang II/Ang I showed an opposite pattern, with progressive reductions significantly linked to worsening respiratory function (e-Table 3; e-Table 6; Fig. 2).

### Markers of RAS activation parallels dynamics of endothelial injury indicators

To investigate whether RAS activation reflects dysregulated endothelial function, we examined the trajectories of RAS peptides in parallel with markers of endothelial injury, including ADMA and EASIX. Among these, ADMA exhibited a trajectory like RAS activation markers, characterized by progressively higher levels in patients who worsened compared with those who remained stable. At D0, ADMA levels did not differ significantly between groups; however, by D3, they were significantly higher in the worsening group (0.72 [IQR 0.62–0.87] vs. 0.61 [IQR 0.50–0.70] µM; *p* < 0.001) (Table 2). Consistent with this pattern, ADMA correlated significantly with RAS peptides at both timepoints, except for Ang 1–7, which correlated with ADMA only at D3 (e-Fig. 3). Similarly, EASIX values were comparable across groups at D0 (*p* = 0.22) but significantly higher in the worsening group at D3 (*p* = 0.01). In ROC analysis, ADMA at D0 demonstrated limited discriminative power for clinical severity (AUC = 0.66), but this improved by D3 (AUC = 0.77) (Table 3). In linear adjusted regression models, increasing ADMA concentrations over time were associated with progressive respiratory deterioration, as indicated by a significant interaction between patient group and ADMA level at D0 (e-Table 3; e-Table 6; Fig. 2).

### Association of RAS markers and endothelial injury indicators with 28-day status

In the partial proportional odds models, 28-day clinical status was significantly associated with both D0 and D3 levels of renin and Ang II/Ang I, but only with D3 levels of Ang I and Ang 1–7. No statistically significant association was observed between Ang II and 28-day clinical status. Overall, angiotensin levels demonstrated a positive association with disease severity, whereas the Ang II/Ang I showed an inverse relationship, with higher levels being predictive of a more favorable 28-day outcome. Among endothelial markers, only baseline ADMA concentrations were predictive of 28-day status, with elevated D0 plasma levels being associated with worse clinical outcomes (Table 4 and e-Table 5; e-Fig. 2). Findings were consistent when RAS inhibitor chronic use was added to the models, confirming the robustness of the main results (e-Table 4 and e-Table 4-bis).

**Table 4:** Odds ratios for 28-day clinical status

			Day 0					Day 3		
Marker	OR	95% CI	*p*-value	SD	BH	OR	95% CI	*p*-value	SD	BH
Renin	1.67	1.15–2.43	0.007	18.06	1	1.91	1.27–2.87	0.002	37.53	1
AngI	1.17	0.83–1.66	0.350	214.57	0	1.88	1.35–3.02	0.001	554.45	1
AngII	1.22	0.86–1.73	0.250	13.99	0	1.26	0.86–1.84	0.220	10.57	0
AngII/I	0.63	0.42–0.94	0.002	0.07	1	0.51	0.33–0.78	0.002	0.05	1
Ang1–7	1.12	0.78–1.63	0.520	19.22	0	1.99	1.33–3.00	0.001	34.49	1
ADMA	1.84	1.23–2.77	0.003	0.13	1	1.41	0.94–2.11	0.080	0.17	0

Odds ratios (ORs) with 95% confidence intervals (CIs) for the 28-day clinical status by biomarker and timepoint. *BH* Benjamini–Hochberg significance correction. *SD* Standard deviation. BH = 1 indicates statistical significance after BH correction. Biomarkers are standardized so ORs reflect the effect of a one SD increase in biomarker level on the odds of worse clinical status at day 28. Clinical status levels: 1 = deceased or receiving IMV; 2 = hospitalized but not requiring IMV; 3 = alive and discharged from the hospital. Level 3 was the baseline comparison group; higher OR indicates worse clinical status at day

28. Models adjusted for age, sex, CKM syndrome, and SOFA score at inclusion. Ang = angiotensin (reported in pg/ml). *ADMA* = asymmetric dimethylarginine (reported in μM/L). Renin reported in pg/ml. *Ang II/I* = calculated as the ratio of angiotensin II to angiotensin I concentration

## Discussion

In this study, we analyzed the trajectories of RAS components and markers of oxidative stress in relation to changes in respiratory status among hospitalized patients with COVID-19. Our principal findings are as follows:

- Patients who experienced respiratory deterioration exhibited delayed activation of the RAS, with increasing levels of renin and Ang I over time.
- RAS activation was accompanied by a shift toward the alternative pathway, evidenced by a progressive decline in the Ang II/Ang I and a corresponding rise in Ang 1–7.
- These changes were associated with elevated circulating ADMA levels, suggesting a simultaneous disturbance in systemic oxidative stress.

This study adds to the existing literature by validating the association between RAS metabolite kinetics and relevant clinical outcomes [29]. Numerous observational studies have demonstrated the predictive value of renin fluctuations for mortality and major adverse kidney events in critically ill patients [30–34]. From a pathophysiological standpoint, renin secretion is triggered by hypotension and reduced organ perfusion, supporting its potential role as a marker of vascular dysfunction [35]. Consistently, we observed that time-dependent variations in serum renin correlated with circulating ADMA, reinforcing the hypothesis that RAS activation may reflect endothelial injury. Similar dynamics were described in a recent study reporting late elevation of renin and endothelial markers in patients with severe COVID-19 [25]. Contrary to the initial hypothesis that SARS-CoV-2 interaction with ACE2 would result in Ang 1–7 depletion [36, 37], our findings showed that Ang 1–7 concentrations increased over the disease course—but only in the worsening group. These patients exhibited a gradual reduction in the Ang II/Ang I, indicative of ongoing loss of ACE function [30]. A relative deficiency in Ang II is consistent with prior studies reporting reduced ACE activity in the serum of patients with both COVID-19-related and unrelated ARDS [38, 39]. In general, an imbalance in RAS enzyme activity —manifested as elevated Ang I without a corresponding rise in Ang II —is a common feature of several critical conditions, including vasodilatory shock [40, 41]. Potential mechanisms include ACE dysfunction due to endothelial injury and increased Ang II degradation mediated by the release of dipeptidyl peptidase 3 (DPP3) [42–44]. Nevertheless, because ACE2 catalyzes the conversion of Ang II to Ang 1–7, it is also biologically plausible that augmented ACE2 activity contributes to the reduced Ang II/Ang I [45]. Two recent studies found significantly higher circulating ACE2 (sACE2) levels, along with lower ACE levels, in patients with severe COVID-19 compared to controls and those with mild disease [46, 47]. In addition, prior studies have shown a positive correlation between sACE2 concentrations and COVID-19 severity [48, 49]. ACE2 is an interferon-stimulated gene and plays a critical role in early tissue tolerance during respiratory infection [50]. Therefore, in our cohort, the late increase in Ang 1–7 levels among patients with worsening respiratory function may reflect a dysregulated inflammatory response. Alternatively, the shift toward Ang 1–7 production could represent an adaptive mechanism to counter oxidative stress, as suggested by concurrent increases in ADMA. SARS-CoV-2 infection predisposes patients to endothelial injury, as evidenced by microangiopathy and a disproportionately high rate of thrombotic events in severe cases [51–54]. In this context, the recognized relationship between accumulation of ADMA and development of cardiovascular dysfunction further support the role of endothelial injury in the progression of COVID-19 [55, 56].

### Interpretation of mechanistic findings

Taken together, the combined pattern of rising renin and Ang I, a blunted Ang II response, increasing Ang 1–7 concentrations, and higher ADMA levels supports a model in which endothelial dysfunction drives RAS dysregulation in worsening COVID-19. In severe disease, inflammatory and endothelial injury processes may impair ACE activity while promoting ACE2 upregulation and shedding, leading to reduced conversion of Ang I to Ang II and enhanced diversion of Ang II toward Ang 1–7. In parallel, elevated circulating dipeptidyl-peptidase 3 (DPP3)—a protease released during cellular injury that rapidly degrades Ang II—may further contribute to the observed Ang II deficiency despite marked RAS activation. This mechanism explains why renin and Ang I increase markedly in patients with poor outcomes, whereas Ang II does not follow the expected physiological rise. The concurrent elevations of ADMA and EASIX further indicate that oxidative stress and endothelial injury evolve in parallel with RAS imbalance, reinforcing the concept that COVID-19 severity is characterized by a vascular-centric phenotype.

## Limitations

Several limitations of this study must be acknowledged. First, the observational design precludes causal inference regarding the metabolites analyzed. Specifically, we cannot determine whether differences in RAS peptides between groups merely reflect infection severity or contribute mechanistically to disease progression. Evidence from clinical trials showing that RAS-modifying treatments do not alter COVID-19 outcomes supports the former interpretation [57, 58]. Second, we did not measure ACE and ACE2 expression directly, so we cannot exclude the involvement of alternative enzymes in angiotensin processing—such as conversion of Ang I to Ang 1–7 by neprilysin, or Ang II degradation via DPP3 release [59]. Nonetheless, several studies have reported parallel increases in circulating Ang 1–7 and ACE2 activity in patients with severe COVID-19 [46, 47, 60]. Third, elevated Ang 1–7 levels may result from increased cleavage of membrane-bound ACE2 into sACE2 by ADAM17, a protease upregulated in response to pro-inflammatory cytokines [61]. In this study, the absence of direct tissue-level measurements of RAS metabolite limits our ability to distinguish between generalized increases in sACE2 and redistribution from tissue to serum potentially causing local Ang 1–7 deficiency. However, a postmortem study showed increased ACE2 and decreased ACE expression in the lungs of patients with both COVID-19 and non-COVID acute respiratory distress syndrome (ARDS), compared to controls [46].

Fourth, the two-timepoint design captures only early changes up to day 3. Although this interval was selected, because most early deterioration in our cohort occurred within the first 72 h, it does not allow assessment of later clinical trajectories. A further limitation concerns the use of the WHO ordinal scale. Dichotomizing patients into worsening versus non-worsening inevitably reduces granularity; however, large transitions were rare in our cohort, with ~ 10% deteriorating by ≥ 2 levels and over 85% showing changes of only ± 1 point. Modelling every step of the scale would, therefore, have added complexity with limited statistical gain. To address this issue, we performed complementary analysis using ΔWHO as a continuous variable, which yielded results consistent with the primary findings (e-Table 8 and e-Table 8-bis).

Finally, although we used a validated high-precision mass spectrometry method for biomarker analysis, reported reference ranges in the literature vary widely and may depend on sample timing and assay technique [62].

## Conclusions

In summary, our findings suggest that changes in renin levels may serve as a biomarker for clinical outcomes in COVID-19. Among patients experiencing respiratory deterioration, the analysis of RAS peptides and ADMA concentrations identified a phenotype characterized by predominant activation of the alternative RAS pathway and evidence of oxidative stress. Further research is warranted to determine the clinical utility of this phenotype and its relevance to trial design and personalized treatment strategies.

## Supplementary Information

Additional file1

## Data Availability

After publication, the data will be made available to others on reasonable requests to the corresponding author. A proposal with detailed description of study objectives and statistical analysis plan will be needed for evaluation of the reasonability of requests. De-identified participant data will be provided after approval from the corresponding author.

## Abbreviations

RAS: Renin–angiotensin system
Ang II: Angiotensin II
Ang I: Angiotensin I
ACE: Angiotensin-converting enzyme
ACE2: Angiotensin-converting enzyme 2
Ang 1–7: Angiotensin 1–7
ADMA: Asymmetric dimethylarginine
RT-PCR: Real-time reverse transcriptase-polymerase
STROBE: Strengthening the Reporting of Observational Studies in Epidemiology
D0: Day 0 (at enrolment)
D3: Day 3
UPLC–MS/MS: Ultraperformance liquid chromatography coupled with tandem mass spectrometry
Ang II/I: Ratio of angiotensin II to angiotensin I
CKM: Cardiovascular–kidney–metabolic syndrome
EASIX: Endothelial Activation and Stress Index
WHO: World Health Organization
MICE: Multiple Imputation by Chained Equations
PMM: Predictive mean matching
SOFA: Sequential Organ Failure Assessment
HC3: Heteroskedasticity robust
ROC: Receiver operating characteristic
IMV: Invasive mechanical ventilation
SD: Standard deviation
BH: Benjamini–Hochberg
FDR: False discovery rate
ICU: Intensive care unit
IQR: Interquartile range
AUC: Area under curve
DPP3: Release of dipeptidyl peptidase 3
sACE2: Circulating ACE2
ARDS: Acute respiratory distress syndrome
