# Supplementary material for: Renin–angiotensin system activation and oxidative stress in hospitalized COVID-19 patients: a single-centre prospective observational study
Source: Intensive Care Med Exp. 2026 Feb 10;14:15. doi: 10.1186/s40635-026-00857-w (PMC12886697; doi:10.1186/s40635-026-00857-w)
Supplement: Supplementary file 1 — Additional file1 [file 40635_2026_857_MOESM1_ESM.docx]

SUPPLEMENTARY MATERIAL

**Ethics Statement**

Samples were collected during the second surge of the COVID-19 pandemic. The study was approved by the Ethical Committee of the XXX (approval number XXX; protocol number XXX, issued on April 2021). All patients, or their legal representatives where applicable, provided written informed consent prior to inclusion in the study, in accordance with national law and the Declaration of Helsinki. All data were prospectively recorded in an electronic database and subsequently anonymized. Research blood samples were collected in coordination with clinically indicated blood draws.

**Clinical and Demographic Variables**

A total of 204 consecutive patients were screened between April and September 2021. Of these, 49 were excluded, leaving 155 patients eligible for analysis (Figure E1). Patient demographics — age, gender, body mass index (BMI), timing of symptom onset, medical history — were recorded at study entry (Table E1). Data on hospital treatments, the Sequential Organ Failure Assessment (SOFA) score, and laboratory variables — including albumin, lymphocyte count and percentage, platelet count, lactate dehydrogenase (LDH), creatinine, D-dimer, C-reactive protein (CRP), procalcitonin, and white blood cell (WBC) count — were collected on the day of sampling. RAS medication status was defined as treatment with a pharmacologic RAS inhibitor at the time of hospitalization (Table 1). We categorized patients by respiratory support level according to their WHO scale (1). Non- pulmonary organ dysfunctions included acute kidney injury (AKI) and septic shock. AKI was operationalized as renal impairment of ≥ Stage 2 according to the Kidney Disease Improving Global Outcomes (KDIGO) creatinine criteria. When pre-hospital baseline creatinine values were unavailable, they were estimated using the Modification of Diet in Renal Disease (MDRD) equation,

as recommended by KDIGO guidelines (2). Septic shock was defined according to the Sepsis-3 criteria (3). Given recent literature highlighting the role of metabolic and cardiorenal dysfunction in COVID-19 severity, we included cardiovascular–kidney–metabolic (CKM) syndrome as a composite comorbidity (4,5). CKM syndrome encompasses individuals at risk for cardiovascular disease (CVD) due to metabolic risk factors (e.g., overweight/obesity with BMI ≥25 kg/m², hypertension, diabetes, or chronic kidney disease), as well as patients with existing CVD (e.g., coronary heart disease, heart failure, stroke, peripheral artery disease, or atrial fibrillation) likely driven by metabolic dysregulation

(6). As markers of endothelial dysfunction, we evaluated asymmetric dimethylarginine (ADMA) and the Endothelial Activation and Stress Index (EASIX), both measured on the day of study sampling. Substantial evidence indicates that elevated ADMA levels are associated with impaired endothelial function in patients with cardiovascular conditions. It is well established that oxidative stress promotes ADMA synthesis and inhibits its degradation (7). Furthermore, recent studies suggest that ADMA may serve as a prognostic biomarker in COVID-19 (8). The EASIX was calculated using the formula: EASIX =LDH (U/L) × creatinine (mg/dL) ÷ platelet count (10⁹/L) (9). The components of EASIX are surrogate markers of endothelial dysfunction, with their values reflecting the severity of endothelial impairment. Notably, higher EASIX values at hospital admission have been associated with a more severe course of COVID-19 (10).

## ARDS Classification and Its Role in the Analysis

Acute respiratory distress syndrome (ARDS) was defined according to the Berlin definition (11), requiring: (1) acute onset of respiratory symptoms, (2) bilateral pulmonary opacities on chest imaging not fully explained by cardiac failure or fluid overload, and (3) impaired oxygenation defined by a PaO₂/FiO₂ ratio ≤300 mmHg (Table E1). For patients treated with invasive mechanical ventilation (IMV) or non-invasive ventilation (NIV), the minimum requirement of PEEP ≥5 cm H₂O was inherently met. In line with recent literature suggesting that patients on high-flow nasal cannula (HFNC) can meet clinical and radiologic criteria for ARDS despite the absence of controlled PEEP,

we also classified HFNC-treated patients as having ARDS if they had a PaO₂/FiO₂ ≤300 mmHg and bilateral infiltrates on imaging (12). While ARDS status was described for eligible patients at both time points (D0 and D3), the primary stratification of clinical severity in this study was based on changes in the WHO ordinal scale, which allowed for comprehensive assessment across the entire cohort regardless of respiratory support modality.

## Patients' trajectories of ventilatory support levels over the first three days from admission

The trajectory of ventilatory support levels over the study period is shown in Figure 1. At baseline (D0), 27 patients (17.4%) did not require any form of oxygen supplementation. Among those receiving respiratory support at inclusion, 90 patients (58.1%) were treated with conventional oxygen therapy (COT), 6 (3.9%) with high-flow nasal cannula (HFNC), 27 (17.4%) with non-invasive mechanical ventilation (NIMV), and 5 (3.2%) were intubated. Between D0 and D3, 89 patients progressed to a lower WHO ordinal scale level and were categorized in the worsening group, while 52 of 66 patients in the non-worsening group maintained stable respiratory status. By D3, a total of 41 patients (27%) required IMV. Among them, 24 (16%) had been on NIMV at inclusion, 11 (7%) on COT, and 2 (1.3%) were not receiving oxygen therapy at baseline.

**Rationale for Day 3 Assessment**

COVID-19 respiratory deterioration often occurs between days 5 and 10 of illness; however, our objective in this study was to evaluate the early dynamics of RAS activation and oxidative stress following hospital admission. For this reason, we selected Day 3 (approximately 72 hours after baseline) as a biologically and clinically meaningful time point that reflects the initial in-hospital phase, before later complications or therapeutic interventions might obscure early physiological changes. Importantly, Day 3 was not chosen arbitrarily. In our cohort, 89 patients (≈60%) had already met the definition of clinical worsening by Day 3, indicating that this interval was sufficiently sensitive to detect meaningful early deterioration. Day 3 therefore represents the period when early respiratory decline typically emerges and when therapeutic decisions—such as the initiation of corticosteroids, antivirals, or escalation of respiratory support—are often made. We thus considered Day 3 an appropriate interval to relate early biomarker dynamics to clinically relevant short-term outcomes. We acknowledge that the two-time-point design limits our ability to characterize longer-term trajectories across the full course of COVID-19, and this limitation is explicitly reported in the main manuscript.

**Pre-analytical Conditions and Hemodynamic Context for Renin and RAS Peptide Measurements**

Renin and angiotensin peptides can be influenced by posture, recent activity, and other pre-analytical factors under controlled outpatient conditions. However, such standardized procedures (e.g., fixed resting periods, upright or supine positioning, minimization of movement) are not feasible or routinely applied in hospitalized or critically ill patients. In this study, blood sampling was performed under clinically stable and comparable hemodynamic conditions, with patients generally resting in bed and without ongoing procedures, fluid boluses, or acute hemodynamic interventions at the time of collection. This timing minimized rapid fluctuations associated with acute circulatory changes and reflects the practical standards applied in acute-care biomarker research. This approach aligns with previous studies assessing renin and RAS activation in critically ill adults, where outpatient-style pre-analytical controls cannot be implemented. Notably, renin has been shown to exhibit stable kinetics over time in critically ill patients and to serve as a marker of tissue perfusion and illness severity rather than a rapidly varying physiological parameter (Gleeson et al., Crit Care Med 2019). Similar stability and prognostic utility of renin and RAS peptides were observed in critically ill patients with COVID-19 (Krenn et al., Ann Intensive Care 2025). Collectively, these data support the validity of renin and RAS peptide measurements obtained during routine clinical care in hospitalized patients, and indicate that the within-patient changes observed in our study are unlikely to be driven by uncontrolled pre-analytical or hemodynamic variability.

**Plasma collection and processing**

Biomarkers quantification was performed on blood samples collected in dedicated tubes.

For renin determination blood samples were collected in VACUETTE® TUBE 1 ml K3E K3EDTA (Greiner Bio-One S.r.l, Cassina de Pecchi, Italy). Tubes were centrifuged at 4000 x g for 5 minutes. For ADMA determination plasma samples were collected through 4 mL Lithium-Heparin Vacuette tubes (Greiner Bio-One S.r.l, Cassina de Pecchi, Italy). Tubes were centrifuged at 4000 x g for 5 minutes.

For angiotensin peptides determination plasma samples were collected through 4 mL EDTA Aprotinin Vacuette tubes (Greiner Bio-One S.r.l, Cassina de Pecchi, Italy) and immediately centrifuged at 2000 × g for 15 minutes at 20 °C.

The supernatant plasma was then aliquoted into pre-labeled tubes and stored at −80 °C until analyses.

## Renin

The determination of direct renin on EDTA plasma was performed using an automated CLIA assay for quantitative analysis on a Liaison XL platform (Diasorin, Italy).

## Asymmetric dimethyl arginine (ADMA)

ADMA was measured in plasma by ultraperformance liquid chromatography/tandem mass spectrometry (UPLC-MS/MS). The UPLC-MS/MS system consisted of an UPLC and autosampler ExionLC AD system (ABSciex, Framingham, MA, USA) and a Qtrap 6500+ (ABSciex, Framingham, MA, USA) equipped with electrospray ion source. Analyses were conducted in positive ion mode. Ammonium formate and ADMA (NG, NG-dimethylarginine dihydrochloride) were purchased from Sigma (Sigma-Aldrich, St. Louis, MO, USA). ADMA: HCl:XH2O (2,3,3,4,4,5,5-D7) ([2H7] ADMA, internal standard) was purchased from Cambridge Isotope Laboratories (Cambridge Isotope Laboratories, Andover, MA, USA). Water, Acetonitrile, Methanol and formic acid (LC-MS grade) were purchased from Biosolve (Biosolve Chimie, Dieuze, France). Stock solutions of ADMA (5 mM) and [2H7] ADMA (5 mM) were prepared in water and stored at -80 °C. The analytical procedure requires a deproteinization step, performed by adding 300 µL of methanol containing 0.3 µmol/L of [2H7] ADMA to 100 µL of plasma sample. After vigorous agitation, the sample was centrifuged at 14 000 x g for 7 minutes at room temperature, then the supernatant was transferred into a vial and injected into UPLC system for analysis. Calibration curve was prepared in water with the

following concentrations: 0.0, 0.15, 0.25, 0.75, 1.0, 1.5 and 5.0 µM. Quality controls were prepared by spiking pooled plasma sample with standard solutions as reported in Table A:

**Table A** Spike concentration for ADMA determination

**ADMA** (µM) L1 -

L2 +0.5

L3 +1.5

Calibration points were treated according to the protocol and calibration curves were constructed by plotting peak area ratio (Analyte/IS) vs. nominal concentration. Samples will be loaded onto a LUNA HILIC column, 3 µm, 200 Å, 100 x 2.0 mm (Phenomenex, Torrance, California, USA). The chromatographic separation was performed with a gradient of mobile phase A (H2O containing 0.1% formic acid and 20 mM ammonium formate) and mobile phase B (acetonitrile), with a flow rate of

0.400 mL/min. The gradient followed this pattern: 0-0.5 min 99% B, 0.5-3.0 min 80% B, 3.0-5.0 min

60% B, 5.0-5.1 min 40% B, 5.1-6.1 min 40% B, 6.1-6.11 min 99% B, 6.11-9.0 min 99% B. The oven

temperature was set at 40 °C. The injection volume was 10 µL, and the total analysis time was 9.0 min. The optimized parameters for the ion source were: temperature at 400 °C, curtain gas at 30, nebulizing gas (GS1) at 30, drying gas (GS2) at 30, collision activated dissociation gas at medium, ion-spray voltage at 4000 V. The multiple reaction monitoring (MRM) transitions for each analyte, their respective collision energy, and cone voltage values were reported in Table B:

**Table B** Instrumental parameters: MS/MS transitions for MRM mode. Compound dependent analytical parameters: DP (declustering potential), EP (entrance potential), CE (collision energy) and CXP (collision cell exit potential)

| **Compound** | **Q1 Mass (Da)** | **Q3 Mass (Da)** | **DP**  **(volts)** | **EP**  **(volts)** | **CE**  **(volts)** | **CXP**  **(volts)** |
| --- | --- | --- | --- | --- | --- | --- |
| ADMA | 203.3 | 46 | 60 | 13 | 30 | 18 |
| [^2^H7] ADMA | 210.2 | 46 | 60 | 13 | 30 | 18 |

Data acquisition was carried out using the mass spectrometer software (Analyst Software 1.7.1, ABSciex, Framingham, MA, USA) while for quantitative analysis was used the processing software (Sciex OS, ABSciex, Framingham, MA, USA).

## Angiotensin peptides

Angiotensin peptides are hormone peptides part of the renin-angiotensin system (RAS). A panel Angiotensin I (AI), Angiotensin II (AII) and Angiotensin 1-7 (A 1-7) were measured in plasma by ultraperformance liquid chromatography/tandem mass spectrometry (UPLC-MS/MS). The UPLC- MS/MS system consisted of an UPLC and autosampler ExionLC AD system (ABSciex, Framingham, MA, USA) and a Qtrap 6500+ (ABSciex, Framingham, MA, USA) equipped with electrospray ion source. Analyses were conducted in positive ion mode. Angiotensin I (Angiotensin I human acetate salt hydrate), Angiotensin II (Angiotensin II human) and Angiotensin 1-7 (Angiotensin Fragment 1- 7 acetate salt hydrate) were purchased from Sigma (Sigma-Aldrich, St. Louis, MO, USA). Angiotensin I (13C6 and 15 N, internal standard) and Angiotensin II (13C6 and 15N, internal standard) were purchased from AnaSpec (AnaSpec, Fremont, CA, USA). Water, acetonitrile, methanol and formic acid (LC-MS grade) were purchased from Biosolve (Biosolve Chimie, Dieuze, France). Standard stock solution of AI (1 mg/mL), AII (1 mg/mL), A1-7 (1 mg/mL), AI 13C6,15N (0.1 mg/mL) and AII 13C6,15N (0.1 mg/mL) were prepared in a solution 25% acetonitrile in water with 0.1% of formic acid and stored at -80 °C. The analytical procedure requires a deproteinization step, performed by adding in a protein LoBind Eppendorf (Eppendorf, SE, Hamburg, Germany) 300 µL of methanol containing 1 ng/mL of 13C6,15N AI and 1 ng/mL of 13C6,15N AII to 100 µL of

plasma sample. After vigorous agitation, the sample was centrifuged at 14000 x g for 7 minutes at

room temperature, then the supernatant was transferred into a vial and injected into UPLC system for analysis. Calibration curve was prepared in a solution of hydrochloridric acid 0.1 M with the following concentrations: AI (0, 100, 150, 300, 600, 750, 1250 and 2500 pg/mL), AII (0, 25, 75, 150,

300, 750, 1250 and 2500 pg/mL) and A1-7 (0, 10, 75, 150, 300, 750, 1250 and 2500 pg/mL).

Quality controls were prepared by spiking pooled plasma sample with standard solutions as reported in Table C:

**Table C** Spike concentration for Angiotensin panel determination

|  | Angiotensin I | Angiotensin II | Angiotensin 1-7 |
| --- | --- | --- | --- |
|  | (pg/mL) | (pg/mL) | (pg/mL) |
| L1 | - | - | - |
| L2 | 500 | 500 | 500 |
| L3 | 1000 | 1000 | 1000 |

Calibration points were treated according to the protocol and calibration curves were constructed by plotting peak area ratio (Analyte/IS) vs. nominal concentration. For Angiotensin 1-7 the calibration curve was constructed by plotting Analyte Area vs. nominal concentration. Samples will be loaded onto a Kinetex 1.7 µm C18, 100 Å, 50 x 2.1 mm (Phenomenex, Torrance, California, USA), coupled with a KrudKatcher ULTRA UPLC In-Line Filter (Phenomenex, Torrance, California, USA).

The chromatograhpic separation was performed with a gradient of mobile phase A (H2O containing 0.2% formic acid) and mobile phase B (acetonitrile containing 0.2% formic acid). The gradient followed this pattern: 0-1.5 min 5% B (0.450 mL/min), 1.5-4.5 min 25% B (0.450 mL/min), 4.5-4.6

min 98% B (0.450 mL/min), 4.6-6.0 min 98% B (0.700 mL/min), 6.0-6.1 min 5% B (0.450 mL/min), 6.1-6.5 min 5% B (0.450 mL/min). The oven temperature was set at 40 °C. The injection volume was 20 µL, and the total analysis time was 6.5 min. The optimized parameters for the ion source were: temperature at 500 °C, curtain gas at 20, nebulizing gas (GS1) at 50, drying gas (GS2) at 50, collision

activated dissociation gas at medium, ion-spray voltage at 5000 V. The multiple reaction monitoring (MRM) transitions for each analyte, their respective collision energy, and cone voltage values were reported in Table D:

**Table D** Instrumental parameters: MS/MS transitions for MRM mode. Compound dependent analytical parameters: DP (declustering potential), EP (entrance potential), CE (collision energy) and CXP (collision cell exit potential)

| **Compound Q1 Mass** | | **Q3 Mass** | **DP** | **EP** | **CE** | **CXP** |
| --- | --- | --- | --- | --- | --- | --- |
|  | **(Da)** | **(Da)** | **(volts)** | **(volts)** | **(volts)** | **(volts)** |
| Angiotensin I | 433.2 | 647.3 | 50 | 10 | 26 | 15 |
| [^13^C615N] Angiotensin I | 435.6 | 654.5 | 50 | 10 | 26 | 15 |
| Angiotensin II | 349.7 | 371.03 | 30 | 10 | 17 | 16 |
| [^13^C615N] Angiotensin II | 352.2 | 371.3 | 50 | 10 | 17 | 16 |
| Angiotensin 1-7 | 300.8 | 371.2 | 40 | 10 | 15 | 16 |

Data acquisition was carried out using the mass spectrometer software (Analyst Software 1.7.1, ABSciex, Framingham, MA, USA) while for quantitative analysis the processing software was used (Sciex OS, ABSciex, Framingham, MA, USA).

# STATISTICAL ANALYSIS

## Missing Data

All patients had blood samples collected, and clinical data recorded on the day of enrolment (D0). Table E2 presents the proportion of data collected over time for the overall cohort. No patients were enrolled without a D0 blood draw for biomarker measurement. Patients with more than 10% missing data were excluded. Additionally, clinical variables with more than 10% missing values were removed from the analysis. For the remaining variables, we examined the mechanism of missingness in those with more than 5% missing values. Biomarker data were most commonly unavailable due to missing laboratory results, supporting the assumption that the data were Missing Completely At Random (MCAR). However, logistic regression models were run to examine whether any of the variables in the data file predict missingness. Respiratory status was significantly associated with

missingness of ADMA at both time points, suggesting a Missing At Random (MAR) mechanism in this case rather than MCAR. We then generated imputations for missing data using the Multiple Imputation by Chained Equations (MICE) algorithm (13). To account for non-normally distributed continuous variables, we employed predictive mean matching (PMM). This technique identifies subjects without missing data on the target variable whose linear predictors are closest to that of the subject with missing data. One subject is randomly selected from among these nearest neighbors, and their observed value is used as the imputed value for the subject with missing data. The available literature suggests that selecting the 10 closest subjects without missing data yields good performance

(14). Analyses conducted under complete-case condition produced similar results (Table E3).

## Respiratory Status Variable

~~Respiratory status was assessed on Days 0, 3, 7 and 28 using the WHO ordinal scale, which defines acuity levels as follows:~~ Respiratory status was assessed on Days 0, 3, 7, and 28. To classify respiratory support in a manner consistent with several recent COVID-19 biomarker studies, we used a modified 6-level respiratory support scale derived from the WHO clinical progression framework, in which the coding direction is reversed compared with the standard WHO Ordinal Scale. In this modified version, higher values indicate less respiratory support (better clinical status) and lower values indicate more intensive support. The six acuity levels used in the present study were defined as follows:

1. Deceased;
2. intubation and invasive mechanical ventilation (IMV);
3. non-invasive mechanical ventilation (NIMV) or high-flow nasal cannula (HFNC);
4. hospitalized with conventional oxygen therapy (COT);
5. hospitalized without oxygen supplementation;
6. alive and discharged from the hospital.

On Day 3, patients were classified into the worsening group if their WHO ordinal scale level was lower than on Day 0. Conversely, those whose Day 3 scale level was equal to or higher than their Day 0 level were assigned to the non-worsening group.

## Clinical Status Day 28

By Day 28, all but 15 patients were classified into one of three conditions: deceased, hospitalized without supplemental oxygen, or alive and discharged from the hospital. Of the remaining 15 patients,

10 were alive and receiving invasive mechanical ventilation (IMV), four were receiving conventional oxygen therapy (COT), and one required non-invasive mechanical ventilation (NIMV). All 10 patients on IMV eventually died, while the five not requiring IMV survived to discharge. To facilitate analysis of 28-day clinical status, patients were categorized into three outcome levels:

1. Deceased or receiving IMV
2. Hospitalized and not requiring IMV
3. Alive and discharged from the hospital

To assess the association between each biomarker (measured at both time points) and 28-day clinical status, we employed generalized ordered logit (gologit) models. This approach enables the fitting of partial proportional odds models by relaxing the parallel-lines assumption only for variables where it is not supported. As a result, gologit models are less restrictive than standard ordered logistic regression (ologit), which often violates this assumption, while remaining more parsimonious and interpretable than non-ordinal alternatives such as multinomial logistic regression (15). Models were adjusted for age, sex, history of CKM syndrome, and SOFA score at admission. Chronic RAS inhibitor use was excluded from the primary analysis for two reasons: (1) the relatively small number of patients in some 28-day status categories increased the risk of unstable estimates when including all candidate covariates; and (2) in univariate analyses with 28-day status as outcome, RAS use showed a weaker association than age, SOFA score, or CKM syndrome (Table E4). To evaluate potential residual confounding, we ran additional models including RAS use as a covariate; results were consistent with the primary analysis, confirming the robustness of the main findings (Table E4- bis). Models were parameterized such that higher odds ratios (ORs) indicate worse 28-day outcomes. To improve interpretability, biomarkers were standardized after removing outliers that disproportionately inflated the standard deviation (SD). ORs therefore reflect the effect of a one-SD increase in biomarker level on the odds of being in a higher severity category at day 28 (Table 4, Figure E2). Standardization was applied only in the partial proportional odds models; linear regression models used raw biomarker values. To address multiple testing across the six primary

biomarkers, the Benjamini–Hochberg (BH) procedure was used to control the false discovery rate (FDR) at 5% (16). *P*-values from all marker-specific models were included in the correction. The BH adjustment did not alter the statistical significance of the findings: markers significant before correction remained significant, and those not significant remained non-significant (Table E5)

## Plasma biomarker levels over the first 3 study days by respiratory trajectory

The primary objective was assessed using baseline-adjusted linear regression models. For each of the six biomarkers analyzed, a separate model was constructed with the plasma biomarker level at D3 as the dependent variable. The D0 biomarker value, SOFA score at inclusion, and age were included as covariates. Patient group (worsening *vs.* non-worsening), sex, chronic use of RAS inhibitors, and history of CKM syndrome were included as categorical variables. To evaluate whether the effect of baseline biomarker concentrations on D3 levels differed according to clinical trajectory, an interaction term between the D0 biomarker value and patient group (worsening vs. non-worsening) was included in the model. This approach was based on the hypothesis that biomarker dynamics vary with the progression of respiratory disease. Because residual diagnostics indicated mild heteroskedasticity and non-normal error distributions, statistical inference for both the main effect of clinical status and the interaction term was obtained from nonparametric pairs bootstrapping (2,000 replications; seed = 12345), which provided empirical standard errors, 95% confidence intervals, and *p*-values (17,18). For comparison, we also estimated heteroskedasticity-robust (HC3) standard errors, which yielded consistent results (Table E6) (19). Variable selection was based on both statistically significant findings in univariate analyses (*p* <0.20) and clinical relevance supported by the literature. The Akaike Information Criterion (AIC) was subsequently used to determine which variables to retain in the final model. To ensure model parsimony, a backward selection process was applied, retaining only those variables that optimized the AIC (20). For the univariate analyses, all models were adjusted for age and sex, which were forced into the models a priori as potential confounders. To account for disease severity, SOFA score was included as a marker of organ dysfunction. CKM

syndrome was incorporated to represent underlying comorbidity burden. In addition, use of RAS inhibitors was retained given its established impact on the renin–angiotensin system and its potential to modify circulating RAS metabolite levels (Table E7). All statistical tests were two-tailed, and a *p*- value <0.05 was considered statistically significant.

**Primary outcome**

Our primary intention in defining “worsening” vs “non-worsening” was not to describe every possible micro-transition of respiratory support, but rather to capture the overall trend of the early respiratory course during hospitalization. In line with this aim, our main models use the D3 biomarker level as the dependent variable and adjust for the corresponding D0 level, age, and SOFA score at inclusion. Statistically, this baseline-adjusted approach targets the net change in biomarker concentration over time, while the worsening/non-worsening grouping separates patients whose clinical course was globally unfavorable from those who were stable or improved. Thus, the binary classification was conceived as a pragmatic way to summarize the early trajectory, rather than to model each specific step of the WHO scale. At the same time, we acknowledge that dichotomization inevitably sacrifices information about the magnitude of change. To evaluate whether biomarker dynamics vary according to the magnitude of respiratory worsening or improvement, we re-estimated all baseline-adjusted models using ΔWHO (continuous) as the clinical predictor, including a ΔWHO × baseline biomarker interaction term. Because more than 85% of transitions ranged from −1 to +1, the ΔWHO variable behaves as a quasi-continuous measure of incremental changes in respiratory support. Across biomarkers, ΔWHO and its interaction with baseline biomarker values consistently predicted D3 biomarker concentrations, indicating that biomarker trajectories reflect the graded degree of clinical deterioration (Table E8). Importantly, we also quantified the distribution of clinical changes to clarify the extent of scale movement in our cohort. Changes exceeding one WHO level were uncommon: approximately 10% of patients deteriorated by ≥2 points, and only about 1% improved by ≥2 points (Table E8-bis). Thus, more than 85% of the cohort experienced either no change or a change of only ±1 point between D0 and D3. This limited frequency of large transitions not only supports our rationale that focusing on the overall trend is clinically meaningful, but also indicates that constructing multiple finely grained ordinal categories would have resulted in sparsely populated cells, potentially introducing model instability and reducing the reliability of ordinal regression estimates.

**References**

1. World Health Organization. WHO R&D Blueprint. Novel Coronavirus. COVID-19 Therapeutic Trial Synopsis. [Internet]. World Health Organization. 2020. Disponibile su: https://[www.who.int/publications/i/item/covid-19-therapeutic-trial-synopsis](http://www.who.int/publications/i/item/covid-19-therapeutic-trial-synopsis)
2. Kellum JA, Lameire N, KDIGO AKI Guideline Work Group. Diagnosis, evaluation, and management of acute kidney injury: a KDIGO summary (Part 1). Crit Care Lond Engl. 4 febbraio 2013;17(1):204.
3. Singer M, Deutschman CS, Seymour CW, Shankar-Hari M, Annane D, Bauer M, et al. The Third International Consensus Definitions for Sepsis and Septic Shock (Sepsis-3). JAMA. 23 febbraio 2016;315(8):801–10.
4. Svensson P, Hofmann R, Häbel H, Jernberg T, Nordberg P. Association between cardiometabolic disease and severe COVID-19: a nationwide case–control study of patients requiring invasive mechanical ventilation. BMJ Open. febbraio 2021;11(2):e044486.
5. Zhou F, Yu T, Du R, Fan G, Liu Y, Liu Z, et al. Clinical course and risk factors for mortality of adult inpatients with COVID-19 in Wuhan, China: a retrospective cohort study. Lancet Lond Engl. 28 marzo 2020;395(10229):1054–62.
6. Ndumele CE, Rangaswami J, Chow SL, Neeland IJ, Tuttle KR, Khan SS, et al. Cardiovascular-Kidney- Metabolic Health: A Presidential Advisory From the American Heart Association. Circulation. 14 novembre 2023;148(20):1606–35.
7. Willeit P, Freitag DF, Laukkanen JA, Chowdhury S, Gobin R, Mayr M, et al. Asymmetric dimethylarginine and cardiovascular risk: systematic review and meta-analysis of 22 prospective studies. J Am Heart Assoc. 28 maggio 2015;4(6):e001833.
8. Sozio E, Hannemann J, Fabris M, Cifù A, Ripoli A, Sbrana F, et al. The role of asymmetric dimethylarginine (ADMA) in COVID-19: association with respiratory failure and predictive role for outcome. Sci Rep. 17 giugno 2023;13(1):9811.
9. Luft T, Benner A, Jodele S, Dandoy CE, Storb R, Gooley T, et al. EASIX in patients with acute graft- versus-host disease: a retrospective cohort analysis. Lancet Haematol. settembre 2017;4(9):e414–23.
10. Kalicińska E, Biernat M, Rybka J, Zińczuk A, Janocha-Litwin J, Rosiek-Biegus M, et al. Endothelial Activation and Stress Index (EASIX) as an Early Predictor for Mortality and Overall Survival in Hematological and Non-Hematological Patients with COVID-19: Multicenter Cohort Study. J Clin Med. 24 settembre 2021;10(19):4373.
11. ARDS Definition Task Force, Ranieri VM, Rubenfeld GD, Thompson BT, Ferguson ND, Caldwell E, et al. Acute respiratory distress syndrome: the Berlin Definition. JAMA. 20 giugno 2012;307(23):2526–33.
12. Ranieri VM, Tonetti T, Navalesi P, Nava S, Antonelli M, Pesenti A, et al. High-Flow Nasal Oxygen for Severe Hypoxemia: Oxygenation Response and Outcome in Patients with COVID-19. Am J Respir Crit Care Med. 15 febbraio 2022;205(4):431–9.
13. van Buuren S, Oudshoorn C. Multivariate Imputation by Chained Equations: MICE V1.0 User’s manual. TNO Report PG/VGZ/00.038 [Internet]. Leiden. Disponibile su: [http://www.multiple-imputation.com](http://www.multiple-imputation.com/)
14. White IR, Royston P, Wood AM. Multiple imputation using chained equations: Issues and guidance for practice. Stat Med. 20 febbraio 2011;30(4):377–99.
15. Williams R. Generalized Ordered Logit/ Partial Proportional Odds Models for Ordinal Dependent Variables [Internet]. The Stata Journal. 2006. Disponibile su: http://www.stata- journal.com/article.html?article=st0097
16. Benjamini Y, Hochberg Y. Controlling the False Discovery Rate: A Practical and Powerful Approach to Multiple Testing. J R Stat Soc Ser B Stat Methodol. 1 gennaio 1995;57(1):289–300.
17. Horowitz JL. Bootstrap methods in econometrics. Annual Review of Economics. 11^a^ ed. 2019;193–224.
18. Pötscher BM, Preinerstorfer D. How reliable are bootstrap-based heteroskedasticity robust tests? Econometric Theory. 39^a^ ed. 2023;789–847horo.
19. Long J, Ervin L. Using heteroscedasticity consistent standard errors in the linear regression model. The American Statistician. 30^a^ ed. 2011;377–99.
20. Venables B, Ripley B. Modern applied statistics with S. In: Springer. 2022.

**e-Figure 1** CONSORT flow chart of the study population up to six months (end of the study)

A total of 204 consecutive patients concern for COVID-19 were admitted to the ED between 15 April to 30 October 2021

N= 34 died

28 in ICU

6 in the medical ward

N =155

included in the analysis

N= 49 excluded 23 SARS-CoV-2 RT-PCR not confirmed

16 were discharged from the Hospital within 48 h from admission 10 refused informed consent

N =122

Discharged alive from the Hospital

**Legend.** ED: emergency department; RT-PCR: reverse transcriptase-polymerase chain reaction; ICU: intensive care unit.

**e-Figure 2. Partial proportional odds models**


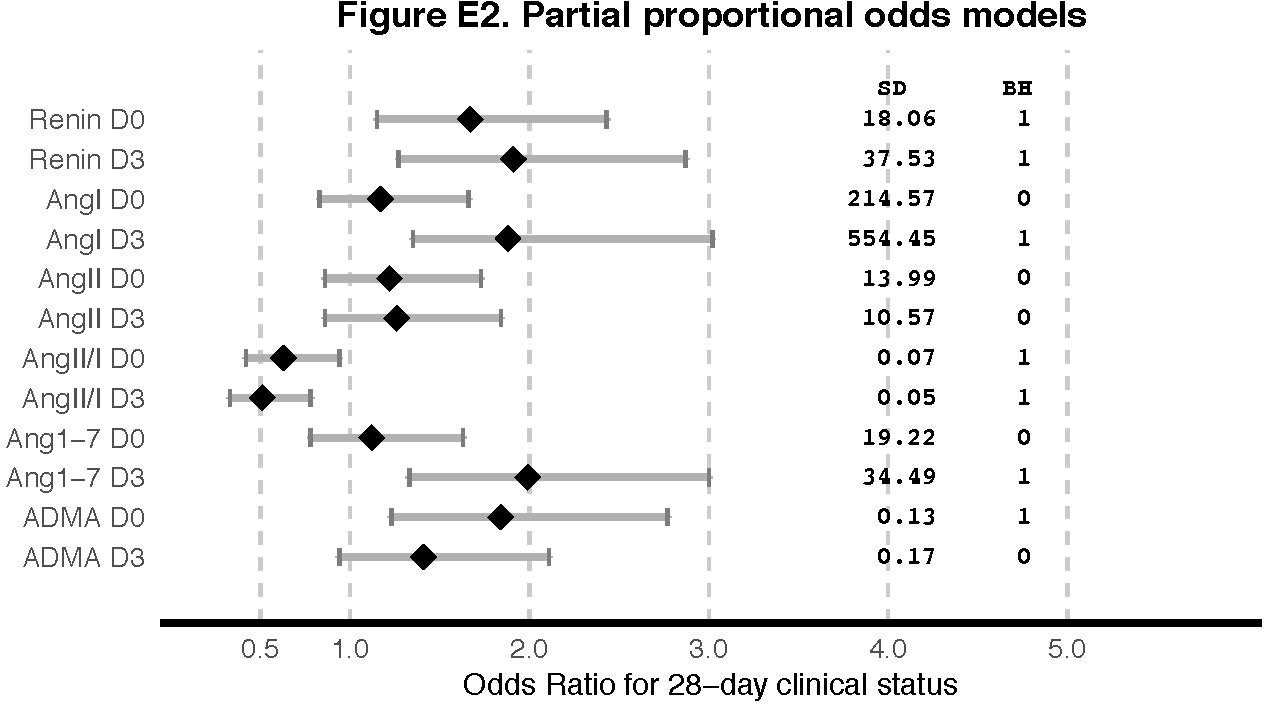


**Legend**. Odds ratios (ORs) and 95% confidence intervals (CIs) for 28-day clinical status by biomarker and time point. Biomarkers were standardized so that the ORs reflect the effect of a one standard deviation (SD) increase in biomarker levels on the odds of being in a higher severity category at day 28. Clinical status levels: 1 = deceased or receiving IMV; 2 = hospitalized but not requiring IMV; 3 = alive and discharged from the hospital. Level 3 of clinical status was considered the baseline comparison group. Accordingly, a higher OR indicates worse clinical status at day 28. Models were adjusted for age, sex, CKM syndrome history, and SOFA score at inclusion. BH = 1 indicates significance after Benjamini-Hochberg correction; BH = 0 indicates non-significance. Ang = angiotensin (reported in pg/ml); Renin reported in pg/ml; ADMA = asymmetric dimethylarginine (reported in μM/L). D0 = day 0; D3 = day 3.

**e-Figure 3.** Correlations between renin-angiotensin system peptides, asymmetric dimethylarginine, and laboratory findings among Participants Over the Study Period (n=155)


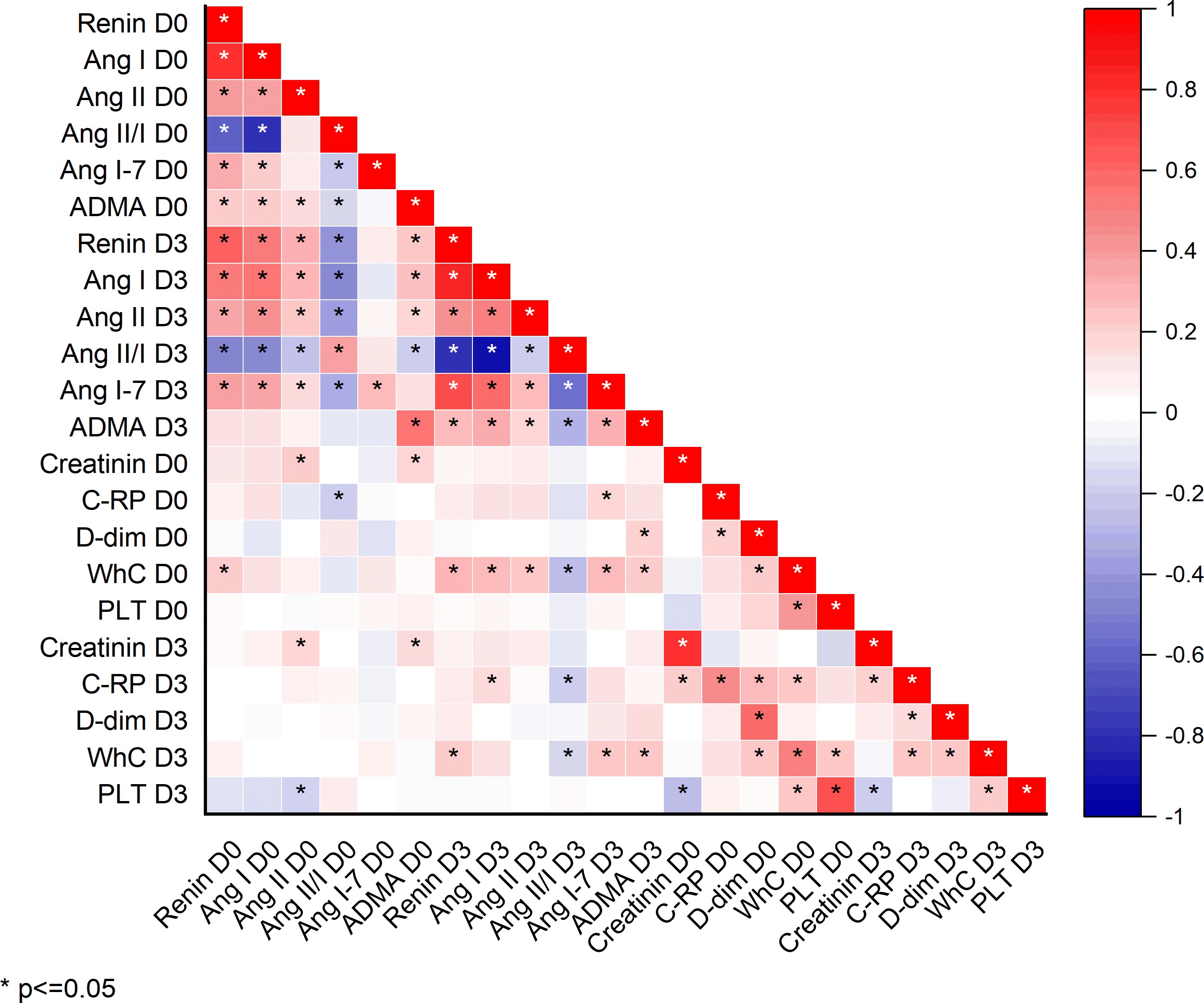


**Legend**. D0 =day 0; D3 =day 3; renin (pg/ml); Ang I =angiotensin I (pg/ml); Ang II =angiotensin II (pg/ml); Ang II/Ang I =angiotensin II/angiotensin ratio; Ang 1-7 =angiotensin 1-7 (pg/ml); ADMA = asymmetric dimethylarginine (µmol/L); Cr =creatinine (mg/dl); C-RP =C-Reactive Protein (mg/dl); D-dim =d-dimer (ng/ml); WBC =absolute White Cell count (10^9^/l); PLT =platelet count (10^9^/l).

^(*)^ *p* <0.05

e-Table 1. Clinical Characteristics by Group

|  | **All**  **(n = 155)** | **Non-wors (n = 66)** | **Wors**  **(n = 89)** | **P-**  **value** |
| --- | --- | --- | --- | --- |
| **Comorbidities** |  |  |  |  |
| CKM syndrome, n (%) | 88 (56.7) | 29 (43.9) | 59 (66.2) | 0.005 |
| Arterial hypertension, n (%) | 56 (36.1) | 14 (21.2) | 42 (47.2) | 0.001 |
| Heart disease, n (%) | 31 (20) | 7 (10.6) | 24 (26.9) | 0.01 |
| Lung disease, n (%) | 7 (4.5) | 3 (4.5) | 4 (4.5) | 0.95† |
| Chronic kidney disease, n (%) | 11 (7.1) | 5 (7.6) | 6 (6.7) | 0.38 |
| Hematologic disease, n (%) | 8 (5.2) | 2 (3) | 6 (6.7) | 0.13† |
| Cancer, n (%) | 15 (9.7) | 4 (6.1) | 11 (12.3) | 0.21† |
| Diabetes mellitus type I, n (%) | 13 (8.4) | 2 (3) | 11 (12.3) | 0.04† |
| Diabetes mellitus type II, n (%) | 25 (16.1) | 9 (13.6) | 16 (17.9) | 0.51 |
| **Hospital Treatments** |  |  |  |  |
| Corticosteroids, n (%) | 140 (90.3) | 57 (83.4) | 83 (90.2) | 0.15 |
| Remdesivir, n (%) | 64 (41.3) | 20 (30.3) | 44 (49.4) | 0.02 |
| Antibiotics, n (%) | 35 (22.6) | 19 (28.8) | 16 (18) | 0.11 |
| **ARDS Day 0** |  |  |  |  |
| Mild, n (%) | 3 (1.9) | 2 (3) | 1 (1.1) | 0.57† |
| Moderate, n (%) | 24 (15.5) | 8 (12.1) | 16 (17.9) | 0.32 |
| Severe, n (%) | 7 (4.5) | 0 (0) | 7 (7.8) | 0.02† |
| **ARDS Day 3** |  |  |  |  |
| Mild, n (%) | 10 (6.4) | 1 (1.5) | 9 (10.1) | 0.04† |
| Moderate, n (%) | 61 (39.3) | 10 (15.1) | 51 (57.3) | <0.001 |
| Severe, n (%) | 17 (10.9) | 0 (0) | 17 (19.1) | <0.001† |

**Legend.** n = number of patients; CKM = cardiovascular-kidney-metabolic; PaO₂ = partial pressure of arterialoxygen; FiO₂ = fraction of inspired oxygen; ARDS = acute respiratory distress syndrome. Statistical comparisons between categorical variables were conducted using the Chi-square test. For variables in which one or more expected cell counts were less than 5, the Fisher’s exact test was used instead, as noted in the table footnotes (†). Wors = worsening group; Non-wors = non-worsening group.

e-Table 2. Proportion of Data Available for Analysis Over Time

|  | | Day 0 |  |  |  | Day 3 |  |
| --- | --- | --- | --- | --- | --- | --- | --- |
| **Marker** | **Missing** |  | **Available** |  | **Missing** |  | **Available** |
| Renin | 8 (5.2%) |  | 147 |  | 9 (5.8%) |  | 146 |
| AngI | 5 (3.2%) |  | 150 |  | 13 (8.4%) |  | 142 |
| AngII | 6 (3.9%) |  | 149 |  | 15 (9.7%) |  | 140 |
| AngII/I | 6 (3.9%) |  | 149 |  | 15 (9.7%) |  | 140 |
| Ang1-7 | 4 (2.6%) |  | 151 |  | 13 (8.4%) |  | 142 |
| ADMA | 16 (10.3%) |  | 139 |  | 17 (11%) |  | 138 |

Legend. Ang = angiotensin; ADMA = asymmetric dimethylarginine; Ang II/I = calculated as the ratio of angiotensin II to angiotensin I concentration.

e-Table 3. Baseline-adjusted OLS (HC3 & Bootstrap), under complete-case analysis

Status and Interaction effects for each marker

| **Outcome** | **Effect** | **Coef (OLS)** | **SE** | **P-value** |  | **SE** | **95% CI** | **P-value** |
| --- | --- | --- | --- | --- | --- | --- | --- | --- |
| Renin | Status | 61.67 | 15.72 | <0.001 |  | 16.76 | [28.82, 95.53] | <0.001 |
| Renin | Interaction | 0.95 | 0.29 | 0.002 |  | 0.3 | [0.3, 1.6] | 0.004 |
| Ang I | Status | 1079 | 378 | 0.005 |  | 330 | [432, 1726] | 0.001 |
| Ang I | Interaction | 0.99 | 0.48 | 0.004 |  | 0.39 | [0.21, 1.77] | 0.012 |
| Ang II | Status | 3.2 | 19.78 | 0.87 |  | 10.36 | [-17.07, 25.56] | 0.75 |
| Ang II | Interaction | 0.19 | 0.59 | 0.74 |  | 0.32 | [-0.43, 0.82] | 0.55 |
| Ang II/I | Status | -0.03 | 0.01 | 0.005 |  | 0.01 | [-0.06, -0.01] | 0.002 |
| Ang II/I | Interaction | -0.3 | 0.1 | 0.004 |  | 0.09 | [-0.49, -0.1] | 0.002 |
| Ang 1–7 | Status | 41.78 | 19.01 | 0.003 |  | 16.67 | [9.1, 74.47] | 0.012 |
| Ang 1–7 | Interaction | 0.94 | 0.39 | 0.019 |  | 0.3 | [0.34, 1.53] | 0.002 |
| ADMA | Status | -0.3 | 0.11 | 0.007 |  | 0.1 | [-0.51, -0.98] | 0.004 |
| ADMA | Interaction | 0.74 | 0.18 | <0.001 |  | 0.17 | [0.39, 1.08] | <0.001 |

**Legend.** OLS = ordinary least squares; Coef = effect estimate from baseline-adjusted OLS regression. Robustness is assessed using HC3 heteroskedasticity correction and nonparametric bootstrap (2,000 reps) for standard error (SE), confidence interval(CI), and P-values. Outcome = marker at Day 3; Status = mean difference at Day 3 between groups (worsening vs non- worsening); Interaction = interaction effect between status and correspondent marker level at baseline (Day 0); Ang = angiotensin; Ang II/I = calculated as the ratio of angiotensin II to angiotensin I concentration; ADMA = asymmetric dimethylarginine.

e-Table 4. Univariate Partial Proportional-Odds

Generalized ordered logit per covariate: p-values and AIC

| **Variable** | **p-value** | **AIC** |
| --- | --- | --- |
| Sofa score | <0.001 | 284.42 |
| CKM syndrome | <0.001 | 292.64 |
| Age | 0.001 | 293.76 |
| RAS inhibitors | 0.09 | 301.82 |
| Sex | 0.28 | 303.47 |

**Legend.** Outcome: 28-day clinical status (ordered, 3 levels). Each row is a separate ordinal logistic model with that single covariate. Lower Akaike Information Criterion (AIC) indicates better fit. SOFA = Sequential Organ Failure Assessment score atinclusion; CKM = cardiovascular-kidney-metabolic; RAS = renin- angiotensin system.

e-Table 4-bis. Odds Ratios for 28-Day Clinical Status from Ordered Logit Models Including RAS Inhibitor Use as a Covariate

|  | | | **Day 0** |  |  |  |  | **Day 3** |  |
| --- | --- | --- | --- | --- | --- | --- | --- | --- | --- |
| **Marker** | **OR** | **95% CI** | **p-value** | **SD** |  | **OR** | **95% CI** | **p-value** | **SD** |
| Renin | 1.64 | 1.13–2.4 | 0.009 | 18.06 |  | 1.78 | 1.18–2.7 | 0.006 | 37.53 |
| Ang I | 1.18 | 0.83–1.68 | 0.35 | 214.57 |  | 1.79 | 1.19–2.71 | 0.005 | 554.45 |
| Ang II | 1.22 | 0.86–1.73 | 0.25 | 13.99 |  | 1.27 | 0.87–1.86 | 0.21 | 10.57 |
| Ang II/I | 0.65 | 0.43–0.97 | 0.03 | 0.07 |  | 0.53 | 0.32–0.86 | 0.01 | 0.05 |
| Ang 1–7 | 1.12 | 0.78–1.63 | 0.51 | 19.22 |  | 2.03 | 1.34–3.06 | 0.001 | 34.49 |
| ADMA | 1.85 | 1.23–2.77 | 0.003 | 0.13 |  | 1.43 | 0.95–2.14 | 0.08 | 0.17 |

Odds ratios (ORs) with 95% confidence intervals (CIs) for 28-day clinical status by biomarker and time point, estimated from ordered logit models adjusted for age, sex, SOFA score, and cardiovascular-kidney-metabolic (CKM) syndrome. Renin- angiotensin system (RAS) inhibitor use was additionally included to assess robustness. Biomarkers were standardized; ORs represent the effect of a one standard deviation (SD) increase in biomarker level on the odds of worse clinical status at day 28. Clinical status categories: 1 = deceased or receiving invasive mechanical ventilation (IMV); 2 = hospitalized, without IMV; 3 = alive and discharged (reference category). Higher ORs indicate worse 28-day status. Units: Renin and angiotensins (Ang) in pg/mL; ADMA = asymmetric dimethylarginine in μM/L; Ang II/I = ratio of angiotensin II to angiotensin I

e-Table 5. BH Correction

| **Marker** | **p-value** | **Rank** | **BH Threshold** | **BH** |
| --- | --- | --- | --- | --- |
| AngI D3 | 0.001 | 1 | 0.004 | 1 |
| Ang1-7 D3 | 0.001 | 2 | 0.008 | 1 |
| AngII/I D3 | 0.002 | 3 | 0.013 | 1 |
| Renin D3 | 0.002 | 4 | 0.017 | 1 |
| AngII/I D0 | 0.002 | 5 | 0.021 | 1 |
| ADMA D0 | 0.003 | 6 | 0.025 | 1 |
| Renin D0 | 0.007 | 7 | 0.029 | 1 |
| ADMA D3 | 0.080 | 8 | 0.033 | 0 |
| AngII D3 | 0.220 | 9 | 0.038 | 0 |
| AngII D0 | 0.250 | 10 | 0.042 | 0 |
| AngI D0 | 0.350 | 11 | 0.046 | 0 |
| Ang1-7 D0 | 0.520 | 12 | 0.050 | 0 |

BH: Benjamini–Hochberg significance correction: BH = 1, the finding is statistically significant after BH correction; BH = 0, the finding is not statistically significant after BH correction. Ang = angiotensin; ADMA= asymmetric dimethylarginine; D0 = day 0; D3 = day 3.

e-Table 6. Baseline-adjusted OLS (HC3 & Bootstrap)

Status and Interaction effects for each marker

| **Outcome** | **Effect** | **Coef (OLS)** | **SE** | **P-value** |  | **SE** | **95% CI** | **P-value** |
| --- | --- | --- | --- | --- | --- | --- | --- | --- |
| Renin | Status | 61.67 | 15.72 | <0.001 |  | 16.76 | [28.82 - 95.53] | <0.001 |
| Renin | Interaction | 0.95 | 0.29 | 0.002 |  | 0.3 | [0.3 - 1.6] | 0.004 |
| Ang I | Status | 1080 | 350 | 0.003 |  | 326 | [439 - 1719] | 0.001 |
| Ang I | Interaction | 0.96 | 0.44 | 0.031 |  | 0.4 | [0.2 - 1.78] | 0.013 |
| Ang II | Status | 3.1 | 19.42 | 0.87 |  | 10.36 | [-17.07 - 25.56] | 0.75 |
| Ang II | Interaction | 0.16 | 0.6 | 0.78 |  | 0.32 | [-0.43 - 3.62] | 0.55 |
| Ang II/I | Status | -0.03 | 0.01 | 0.003 |  | 0.01 | [-0.06 - -0.01] | 0.002 |
| Ang II/I | Interaction | -0.28 | 0.09 | 0.004 |  | 0.09 | [-0.49 - -0.1] | 0.002 |
| Ang 1–7 | Status | 39.36 | 16.67 | 0.021 |  | 16.67 | [9.1 - 74.47] | 0.012 |
| Ang 1–7 | Interaction | 0.94 | 0.32 | 0.004 |  | 0.3 | [0.34 - 1.53] | 0.002 |
| ADMA | Status | -0.3 | 0.1 | 0.004 |  | 0.1 | [-0.51 - -0.98] | 0.004 |
| ADMA | Interaction | 0.74 | 0.17 | <0.001 |  | 0.17 | [0.39 - 1.78] | <0.001 |

Legend. OLS = ordinary least squares; Coef = effect estimate from baseline-adjusted OLS regression. Robustness is assessed using HC3 heteroskedasticity correction and nonparametric bootstrap (2,000 reps) for standard error (SE), confidence interval (CI), and P-values. Outcome = marker at Day 3; Status = mean difference at Day 3 between groups (worsening vs non- worsening); Interaction = interaction effect between status and correspondent marker level at baseline (Day 0); Ang = angiotensin; Ang II/I = calculated as the ratio of angiotensin II to angiotensin I concentration; ADMA = asymmetric dimethylarginine

e-Table 7. Univariate OLS

|  | Screening threshold p < 0.2 |  |
| --- | --- | --- |
| **Marker** | **Covariate** | **p-value** |
| ADMA D3 | CKM syndrome | <0.001 |
| ADMA D3 | CPR | 0.423 |
| ADMA D3 | Creatinine | 0.493 |
| ADMA D3 | D-dim | 0.857 |
| ADMA D3 | Interval | 0.708 |
| ADMA D3 | P/F | 0.272 |
| ADMA D3 | RAS inhibitors use | 0.98 |
| ADMA D3 | Sofa | <0.001 |
| ADMA D3 | Scale D0 | 0.433 |
| Ang 1-7 D3 | CKM syndrome | 0.077 |
| Ang 1-7 D3 | CPR | 0.045 |
| Ang 1-7 D3 | Creatinine | 0.703 |
| Ang 1-7 D3 | D-dim | 0.559 |
| Ang 1-7 D3 | Interval | 0.647 |
| Ang 1-7 D3 | P/F | 0.024 |
| Ang 1-7 D3 | RAS inhibitors use | 0.305 |
| Ang 1-7 D3 | Sofa | 0.003 |
| Ang 1-7 D3 | Scale D0 | 0.654 |
| Ang I D3 | CKM syndrome | 0.113 |
| Ang I D3 | CPR | 0.014 |
| Ang I D3 | Creatinine | 0.182 |
| Ang I D3 | D-dim | 0.315 |
| Ang I D3 | Interval | 0.518 |
| Ang I D3 | P/F | 0.908 |
| Ang I D3 | RAS inhibitors use | 0.088 |
| Ang I D3 | Sofa | <0.001 |
| Ang I D3 | Scale D0 | 0.921 |
| Ang II D3 | CKM syndrome | 0.198 |
| Ang II D3 | CPR | 0.127 |
| Ang II D3 | Creatinine | 0.763 |
| Ang II D3 | D-dim | 0.161 |
| Ang II D3 | Interval | 0.088 |
| Ang II D3 | P/F | 0.077 |

**Legend:** Each row shows the p-value from a univariate ordinary least squares (OLS) model (age and sex forced + one covariate). Screening threshold p < 0.20. D3 = day 3; D0= day 0 ; Ang = angiotensin; Ang II/I = ratio of angiotensin II to angiotensin I concentration; ADMA = asymmetric dimethylarginine. Covariates: CKM = cardiovascular-kidney-metabolic; RAS = renin- angiotensin system; Sofa = sofa score at day 0; CPR = c-reactive protein at day 0; Creatinine = creatinine at day 0; Interval = days from onset symptoms to study inclusion; D-dim = D-dimer at day 0, P/F = PaO2/FiO2 ratio at inclusion; Scale D0 = WHO ordinal scale level at day 0.

Table E8. Association Between Change in WHO Ordinal Scale (ΔWHO) and Biomarker Trajectories

| Linear regression models with D3 biomarker as outcome; ΔWHO = WHOD3 − WHOD0 | | | | | | | | | | | | | |
| --- | --- | --- | --- | --- | --- | --- | --- | --- | --- | --- | --- | --- | --- |
|  | |  | | **ΔWHO** | |  | |  |  | | **Interaction** |  | |
| **Biomarker** | | **β** | | **95% CI** | | **p** | |  | **β** | | **95% CI** | **p** | |
| Renin D3 | | −16.52 | | −29.35 to  −4.13 | | 0.010 | |  | −0.51 | | −0.92 to  −0.10 | 0.015 | |
| Ang I D3 | | −295.00 | | −481 to −109 | | 0.002 | |  | −0.41 | | −0.79 to  −0.04 | 0.029 | |
| Ang II / Ang I D3 | | 0.02 | | 0.005 to  0.030 | | 0.005 | |  | 0.22 | | 0.094 to  0.354 | 0.001 | |
| Ang II D3 | | 1.29 | | −2.18 to 4.75 | | 0.464 | |  | −0.23 | | −0.29 to  −0.18 | <0.001 | |
| Ang 1-7 D3 | | −23.33 | | −45.64 to  −1.13 | | 0.039 | |  | −0.68 | | −1.02 to  −0.36 | <0.001 | |
| ADMA D3 | | 0.13 | | −0.01 to 0.27 | | 0.07 | |  | −0.33 | | −0.55 to  −0.01 | 0.005 | |

**Legend.** Models adjusted for baseline biomarker, age, sex, cardiovascular–kidney–metabolic (CKM) syndrome, renin–angiotensin system (RAS) inhibitor use, and SOFA score at inclusion. Ang = angiotensin; D3 = day 3; Ang II / Ang I = angiotensin II to angiotensin I ratio; ADMA = asymmetric dimethylarginine; ΔWHO interpretation: negative values = worsening, positive values = improvement; interaction = interaction term between day 0 biomarker and ΔWHO.

**Table E8 bis. Distribution of ΔWHO Changes Between Day 0 and Day 3**

| ΔWHO = WHOD3 − WHOD0 (N = 155) | | |
| --- | --- | --- |
| ΔWHO Category | N | % |
| Worsened (any) | 89 | 57.4% |
| - Worsened by −1 | 73 | 47.1% |
| - Worsened by −2 | 14 | 9.0% |
| - Worsened by −3 | 2 | 1.3% |
| Stable (0) | 55 | 35.5% |
| Improved (any) | 11 | 7.1% |
| - Improved by +1 | 10 | 6.5% |
| - Improved by +2 | 1 | 0.6% |

**Table E9. Marker Systemic Levels in Patients Not Receiving RAS Inhibitors**

|  |  | **Day 0** |  |  |  | **Day 3** |  |
| --- | --- | --- | --- | --- | --- | --- | --- |
| **Marker** | Non-worsening | Worsening | p-value† |  | Non-worsening | Worsening | p-value† |
| Renin | 13.1 (4.4–28.4) | 13.5 (6.4–36.2) | 0.43 |  | 9.3 (3.8–20.3) | 58.4 (20.5–130) | <0.001 |
| Ang I | 176.5 (100–362) | 250 (103.5–572.2) | 0.10 |  | 175 (100–343) | 958 (616–2358) | <0.001 |
| Ang II | 19 (15–33.5) | 24 (15–34.7) | 0.04 |  | 16 (15–22) | 20 (15–34) | 0.34 |
| Ang II/I | 0.11 (0.06–0.17) | 0.08 (0.04–0.16) | 0.50 |  | 0.11 (0.05–0.15) | 0.02 (0.01–0.03) | <0.001 |
| Ang 1–7 | 36 (21–61) | 15 (10–34) | 0.002 |  | 17 (10–13) | 64.5 (37.5–102) | <0.001 |
| ADMA | 0.54 (0.45–0.61) | 0.59 (0.53–0.78) | 0.03 |  | 0.61 (0.50–0.71) | 0.74 (0.63–1.03) | <0.001 |

**Legend.** The analysis includes a subgroup of 113 patients not receiving RAS inhibitors at inclusion, among whom 57 met the criteria for clinical worsening. RAS = renin–angiotensin system; Renin reported in pg/mL; Ang = angiotensin (pg/mL); Ang II/I = ratio of angiotensin II to angiotensin I concentration; ADMA = asymmetric dimethylarginine (µM/L). Variables are presented as median (interquartile range). † Mann–Whitney test (significance level < 0.05).
